# A Hybrid Model of Mammalian Cell Cycle Regulation

**DOI:** 10.1371/journal.pcbi.1001077

**Published:** 2011-02-10

**Authors:** Rajat Singhania, R. Michael Sramkoski, James W. Jacobberger, John J. Tyson

**Affiliations:** 1 Department of Biological Sciences, Virginia Polytechnic Institute and State University, Blacksburg, Virginia, United States of America; 2 Case Comprehensive Cancer Center, Case Western Reserve University, Cleveland, Ohio, United States of America; Medical College of Wisconsin, United States of America

## Abstract

The timing of DNA synthesis, mitosis and cell division is regulated by a complex network of biochemical reactions that control the activities of a family of cyclin-dependent kinases. The temporal dynamics of this reaction network is typically modeled by nonlinear differential equations describing the rates of the component reactions. This approach provides exquisite details about molecular regulatory processes but is hampered by the need to estimate realistic values for the many kinetic constants that determine the reaction rates. It is difficult to estimate these kinetic constants from available experimental data. To avoid this problem, modelers often resort to ‘qualitative’ modeling strategies, such as Boolean switching networks, but these models describe only the coarsest features of cell cycle regulation. In this paper we describe a hybrid approach that combines the best features of continuous differential equations and discrete Boolean networks. Cyclin abundances are tracked by piecewise linear differential equations for cyclin synthesis and degradation. Cyclin synthesis is regulated by transcription factors whose activities are represented by discrete variables (0 or 1) and likewise for the activities of the ubiquitin-ligating enzyme complexes that govern cyclin degradation. The discrete variables change according to a predetermined sequence, with the times between transitions determined in part by cyclin accumulation and degradation and as well by exponentially distributed random variables. The model is evaluated in terms of flow cytometry measurements of cyclin proteins in asynchronous populations of human cell lines. The few kinetic constants in the model are easily estimated from the experimental data. Using this hybrid approach, modelers can quickly create quantitatively accurate, computational models of protein regulatory networks in cells.

## Author Summary

The physiological behaviors of cells (growth and division, differentiation, movement, death, etc.) are controlled by complex networks of interacting genes and proteins, and a fundamental goal of computational cell biology is to develop dynamical models of these regulatory networks that are realistic, accurate and predictive. Historically, these models have divided along two basic lines: deterministic or stochastic, and continuous or discrete; with scattered efforts to develop hybrid approaches that bridge these divides. Using the cell cycle control system in eukaryotes as an example, we propose a hybrid approach that combines a continuous representation of slowly changing protein concentrations with a discrete representation of components that switch rapidly between ‘on’ and ‘off’ states, and that combines the deterministic causality of network interactions with the stochastic uncertainty of random events. The hybrid approach can be easily tailored to the available knowledge of control systems, and it provides both qualitative and quantitative results that can be compared to experimental data to test the accuracy and predictive power of the model.

## Introduction

The cell division cycle is the fundamental physiological process by which cells grow, replicate, and divide into two daughter cells that receive all the information (genes) and machinery (proteins, organelles, etc.) necessary to repeat the process under suitable conditions [1]. This cycle of growth and division underlies all biological expansion, development and reproduction. It is highly regulated to promote genetic fidelity and meet the demands of an organism for new cells. Altered systems of cell cycle control are root causes of many severe health problems, such as cancer and birth defects.

In eukaryotic cells, the processes of DNA replication and nuclear/cell division occur sequentially in distinct phases (S and M) separated by two gaps (G1 and G2). Mitosis (M phase) is further subdivided into stages: prophase (chromatin condensation, spindle formation, and nuclear envelope breakdown), prometaphase (chromosome attachment and congression), metaphase (chromosome residence at the mid-plane of the spindle), anaphase (sister chromatid separation and movement to opposite poles of the spindle), telophase (re-formation of the nuclear envelopes), and cytokinesis (cell division). G1 phase is subdivided into uncommitted and committed sub-phases, often referred to as G1-pm (postmitotic interval) and G1-ps (pre S phase interval), separated by the ‘restriction point’ [2]. In this paper, we shall refer to the sub-phases G1-pm and G1-ps as ‘G1a’ and ‘G1b’ respectively.

Progression through the correct sequence of cell-cycle events is governed by a set of cyclin-dependent kinases (Cdk's), whose activities rise and fall during the cell cycle as determined by a complex molecular regulatory network. For example, cyclin synthesis and degradation are controlled, respectively, by transcription factors and ubiquitin-ligating complexes whose activities are, in turn, regulated by cyclin/Cdk complexes.

Current models of the Cdk control system can be classified as either continuous or discrete. Continuous models track the changes of protein concentrations, *C_j_*(*t*) for *j* = 1, 2, …, *N*, by solving a set of nonlinear ordinary differential equations (ODEs) of the form:
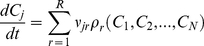
(1)where ρ*_r_* is the rate of the *r* ^th^ reaction and ν*_ir_* is the stoichiometric coefficient of species *i* in reaction *r*. To each rate term is associated one or more kinetic constants that determine exactly how fast the reaction proceeds under specific conditions. These kinetic constants must be estimated from experimental data, and often there is insufficient kinetic data to determine their values. Nonetheless, continuous models, based on rate equations, have been used successfully to account for the properties of cell proliferation in a variety of cell types: yeast [3], [4], [5], fruit fly [6], frog egg [7], [8], and cultured mammalian cells [9], [10], [11]. They have also proved successful in predicting novel cell-cycle characteristics [12], [13].

Discrete models, on the contrary, represent the state of each regulatory protein as *B_j_*(*τ* ) = 0 or 1 (inactive or active), and the state variables update from one discrete time step to the next (*τ* = 0, 1, 2, … = ticks of a metronome) according to the rule:
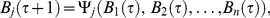
(2)where Ψ*_j_*(…) is a Boolean function (i.e., it equates to either 0 or 1) determined by the topology of the reaction network. For Boolean networks (BNs) there is no notion of reaction ‘rate’ and, hence, no need to estimate kinetic constants. BN models of the Cdk regulatory network have been proposed for yeast cells [14], [15] and for mammalian cells [16]. They have been used to study notions of ‘robustness’ of the cell cycle, but they have not been compared in detail to quantitative properties of cell cycle progression, and they have not been used as predictive tools.

In this paper we propose to combine the strengths of both continuous and discrete modeling, while avoiding the weaknesses of each. Our ‘hybrid’ model is inspired by the work of Li et al. [14], who proposed a BN for cell cycle controls. Their model employs 11 state variables that move around in a space of 2^11^ = 2048 possible states. Quite remarkably they found that 1764 of these states converge quickly onto a ‘super highway’ of 13 consecutive states that represent a typical cell cycle trajectory (G1b—S—G2—M—G1a). The results of Li et al. indicate that the cell cycle control network is ‘robustly designed’ in the sense that even quite large perturbations away from the usual sequence of cell cycle states are quickly restored to the super highway. In the model of Li et al., G1a is a stable steady state; they do not address the signals that drive cells past the restriction point (the G1a-to-G1b transition).

Despite their intuitive appeal, Boolean models have severe limitations. First of all, metronomic time in BN's is unrelated to clock time in the laboratory, so Boolean models cannot be compared to even the most basic observations of time spent by cells in the four phases of the division cycle [1]. Also, these models do not incorporate cell size, so they cannot address the evident importance of cell growth in driving events of the cell cycle [17], [18], [19]. Lastly, cyclins are treated as either absent or present (0 or 1), so Boolean models cannot simulate the continuous accumulation and removal of cyclin molecules at different stages of the cell cycle [20].

Our goal is to retain the elegance of the Boolean representation of the switching network, while introducing continuous variables for cell size, cell age, and cyclin composition, in order to create a model that can be compared in quantitative detail to experimental measurements with a minimal number of kinetic parameters that must be estimated from the data. To this end, we keep the cyclin regulators as Boolean variables but model the cyclins themselves as continuous concentrations that increase and decrease due to synthesis and degradation. Next, we replace the Boolean model's metronome with real clock time to account for realistic rates of cyclin synthesis and degradation, and for stochastic variability in the time spent in each Boolean state of the model. Finally, we introduced a cell size variable, *M*(t), which affects progression through late G1 phase. *M*(t) increases exponentially with time as the cell grows and decreases by a factor of ∼2 when the cell divides. (The assumption of exponential growth is not crucial; similar results are obtained assuming linear growth between cell birth and division.)

Since the pioneering work of Leon Glass [21], [22], hybrid (discrete-continuous) models have been employed by systems biologists in a variety of forms and contexts [23], [24], [25]. Engineers have been modeling hybrid control systems for many years [26], [27], [28], and they have created powerful simulation packages for such systems [29]: SIMULINK [28], SHIFT [30], [31] and CHARON [32], to name a few. We have not used these simulation packages because our model can be solved analytically.

## Results

### Hybrid modeling approach

The modeling approach we are proposing is hybrid in two senses. First, we employ both continuous and discrete variables, and second we allow for both deterministic and stochastic processes. Concerning the components of the control system, we track cyclin levels as continuous concentration variables, but we use discrete Boolean variables to represent the activities (‘on’ or ‘off’) of the regulatory proteins (transcription factors and ubiquitinating enzymes) that control cyclin synthesis and degradation. This distinction is equivalent to a presumed ‘separation of time scales’: the activities of the regulatory proteins change rapidly between 0 and 1, while the concentrations of cyclins change more slowly due to synthesis and degradation. The Boolean variables, we assume, proceed from one state to the next according to a fixed sequence corresponding roughly to the super highway of Li et al. [14]. The time spent in each state, however, is not a ‘tick’ of the metronome but rather the sum of a deterministic execution time (which may be 0) plus a random, exponentially distributed waiting time. In this sense, the model combines deterministic and stochastic processes.

In its present version, our model is not fully autonomous. The discrete variables do not update according to Boolean functions of the current state of the network. Rather, they go through a fixed sequence of states predetermined by the Boolean network model of Li et al. [14]. The discrete variables determine the rates of synthesis and degradation of the continuous variables (the cyclins), and the cyclins feedback on the discrete variables by determining how much time is spent in some of the Boolean states. This strategy keeps the model simple and is appropriate for the cases, considered in this paper, of unperturbed cycling of ‘wild type’ cells, which travel serenely along the super highway of Li et al. To consider more complicated cases, of mutant cells that travel a different route through discrete state space or of cells that are perturbed by drugs or radiation, we will have to elaborate on this basic model with additional rules governing the interactions of the discrete and continuous variables. We are currently working on alternative strategies to adapt this basic modeling paradigm to more complex situations.

**Figure 1:**
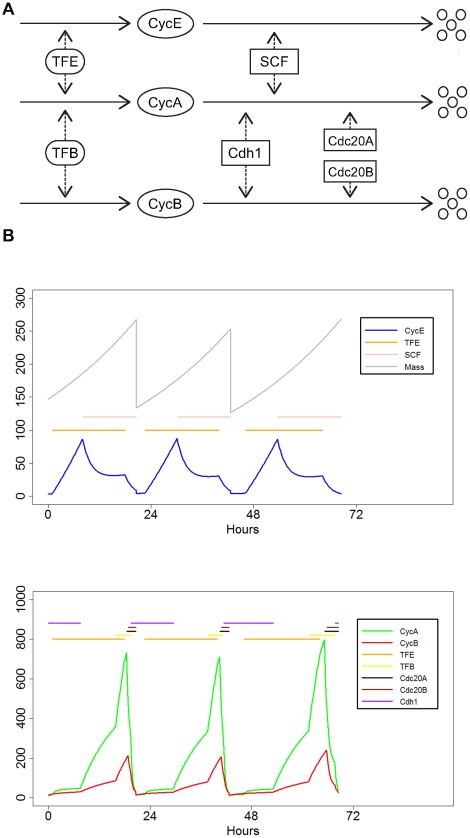
The model. (A) The synthesis and degradation of cyclin proteins is regulated by transcription factors (TFE and TFB) and by ubiquitination machinery (SCF, Cdc20 and Cdh1). (B) Three successive cell cycles are simulated as explained in the Methods. Upper panel: gray curve, 30·*M*(*t*); blue curve, [CycE]·*M*(*t*); the gold line and the pink line indicate the time periods when TFE = 1 and SCF = 1, respectively. Lower panel: green curve, [CycA]·*M*(*t*); red curve, [CycB]·*M*(*t*); the colored bars indicate the time periods when the Boolean variables are active, according to the legend in the inset.

Our model (Fig. 1) tracks three cyclin species (A, B and E), two transcription factors (‘TFE’ and ‘TFB’) and two different E3 ubiquitin-ligase complexes (APC-C and SCF). TFE drives the synthesis of cyclins E and A early in the cell cycle (comparable to the E2F family of transcription factors) [33], and TFB drives the synthesis of cyclins B and A late in the cell cycle (comparable to FoxM1 and Myc) [34], [35]. The Anaphase Promoting Complex—Cyclosome (APC-C) is active during M phase and early G1, when it combines with Cdc20 and Cdh1 to label cyclins A and B for degradation by proteasomes. We make a further distinction between Cdc20 activity on cyclin A (Cdc20A, active throughout mitosis) from Cdc20 activity on cyclin B (Cdc20B, activated at anaphase). The SCF labels cyclin E for degradation via ubiquitination, but only when cyclin E is phosphorylated [36], which we assume is correlated primarily with cyclin A/Cdk2 activity [37].

In our model, the two transcription factors and the four ubiquitination factors are each represented by a Boolean variable, *B*_TFE_, etc. For each cyclin component we write an ordinary differential equation, d[CycX]/d*t* = *k*_sx_−*k*_dx_[CycX], where the rate ‘constants’ for synthesis and degradation, *k*_sx_ and *k*_dx_, depend on the Boolean variables (see Table 1). Hence, each cyclin concentration is governed by a piecewise linear ODE. The parameters in the model (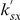, 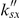, etc.) are assigned numerical values (Table 1), chosen to fit observations of how fast cyclins accumulate and disappear during different phases of the cell cycle.

**Table 1:** Hybrid model of mammalian cell cycle control.

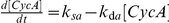	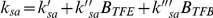	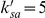	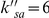	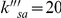
	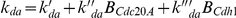	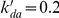	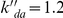	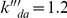
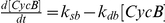	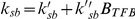	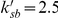	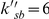	
	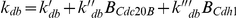	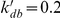	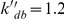	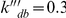
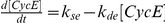				
	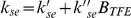 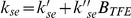	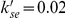	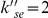	
	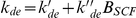	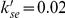	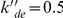	
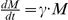	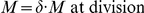	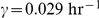	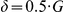 a	
			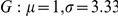	

a *G* is a Gaussian random variable with mean = 1, *σ* = 3.3%.

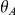
 = 12.5, 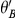 = 21.25, 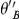 = 3, 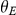 = 80.

Next, we must assign rules for updating the Boolean variables in the model. We assume that the Boolean variables follow a strict sequence of states (see Table 1) that corresponds roughly to the super highway discovered by Li et al. [14]. This sequence of states conforms to current ideas of how the mammalian cell cycle is regulated. Newborn cells are said to be in ‘G1a’ state, because they are not yet committed to a new round of DNA synthesis and mitosis. The transcription factors, TFE and TFB, are silent, and Cdh1/APC-C is active, so the levels of cyclins A, B and E are low in newborn cells. For a mammalian cell to leave the G1a state and commit to a new round of DNA replication and division, it must receive a specific set of extracellular signals (growth factors, matrix binding factors, etc.), which up-regulate the activity of TFE. We assume that these ‘proliferation signals’ are present and that our (simulated) cell spends only a few hours in G1a before transiting into G1b. In our model, the time spent in G1a is an exponentially distributed random variable with mean = 2 h. When the cell passes the ‘restriction point’ and enters G1b, TFE is activated and CycE begins to accumulate. Among other chores, Cdk2/CycE inactivates Cdh1/APC-C, allowing Cdk2/CycA dimers to accumulate. In our model, the transition from early G1b to late G1b is weakly size dependent, because the condition for this transition is that [CycE]*Mass exceeds a certain threshold (*θ*_E_). Because this transition depends on cell mass, those cells that are larger than average tend to make the transition sooner, and cells that are smaller than average tend to make the transition later. This effect allows the cell population to achieve a stable size distribution. In the late G1b state, CycA/Cdk2 level rises to a certain threshold (*θ*_A_), when it triggers entry into S phase. Cdk2/CycA also promotes the degradation of cyclin E by SCF during S phase. We assume that DNA synthesis requires at least 7 h.

Cyclin B begins to accumulate in late G1 and S, after Cdh1 is inactivated, but the major accumulation of cyclin B protein occurs in G2 phase, after DNA synthesis is completed and TFB is activated. The G2—M transition is delayed until enough Cdk1/CycB dimer accumulates ([CycB]>*θ*_B_′) to promote entry into prophase and the appearance Cdc20A/APC-C, which begins the process of cyclin A degradation [38], [39], [40]. Cdc20B/APC-C is activated at the metaphase—anaphase transition, where it promotes three crucial tasks: (1) separation of sister chromatids by the mitotic spindle, (2) partial degradation of cyclin B, and (3) re-activation of Cdh1. Cdh1/APC-C degrades Cdc20 [41], and then finishes the job of cyclin B degradation (telophase). When [CycB] drops below the threshold *θ*_B_″, the cell finishes telophase and divides into two newborn daughter cells in G1 phase (unreplicated chromosomes) with low levels of cyclins A, B and E.

We assume that cell division is symmetric, with some variability; i.e., the mass of the two daughter cells at birth are *δM*_div_ and (1−*δ*)*M*_div_, where *M*_div_ = mass of mother cell at division, and *δ* is a Gaussian-distributed random variable with mean = 0.5 and standard deviation = 0.0167. In all simulations reported here we assume that cells grow exponentially between birth and division. However, we have also simulated linear growth, and the results are not significantly different.

We introduce stochastic effects into the model by assuming that the time spent in each state of the Boolean subsystem, as it moves along the super highway, has a random component (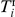) as well as a deterministic component (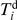): 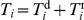. From Table 1, we see that 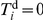 for *i* = 1, 6, 7, 8, and 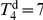 h. For the remaining cases (*i* = 2, 3, 5, 9), 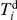 is however long it takes for the cyclin variable to reach its threshold. The stochastic component for each transition is a random number chosen from an exponential distribution with mean = *λ_i_*. The random time delay is calculated from a uniform random deviate, *r*, by the formula 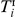 = 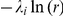. The values chosen for the *λ_i_*'s are given in Table 1.

In the Methods section, we describe how we simulate the progression of a single cell through its DNA replication/division cycle. Because the model's differential equations are piecewise linear, they can be solved analytically, and an entire ‘cell cycle trajectory’ can be determined by computing a few random numbers and solving some algebraic equations. A typical result of such simulations, over three cell cycles, is illustrated in Fig. 1B. Not surprisingly, the accumulation and loss of the cyclins correlate with the activities of the cyclin regulators. At the beginning of each cycle, the cell starts in State 1 (G1a phase in Table 1), with low levels of all cyclin because TFE and TFB are off and Cdh1 is on. When the cell leaves G1a, TFE turns on and cyclin E rises rapidly, but cyclin A increases only modestly, because Cdh1 is still active in early G1b. Cdh1 turns off when cyclin E level crosses *θ*_E_, allowing cyclin A to increase dramatically in late G1b and drive the cell into S phase (State 4). Cyclin B increases modestly in late G1 and S phase, because Cdh1 is off but TFB has not yet turned on. Cyclin E is degraded in S phase, because SCF is now active. When the cell finishes DNA synthesis, TFB turns on, causing further increase of cyclins A and B. When cyclin B level rises above its first threshold, *θ*_B_′, the cell enters prophase (State 6) and then prometaphase-metaphase (State 7). During State 7, cyclin A level drops precipitously because Cdc20A is turned on. After the replicated chromosomes are fully aligned on the mitotic spindle, Cdc20B turns on (State 8) and cyclin B is partially degraded. Cdc20B activates Cdh1 (State 9) and cyclin B is degraded even faster. When cyclin B level drops below its second threshold, *θ*_B_″, the cell divides and returns to G1a (State 1).

### Cyclin distributions in an asynchronous culture

**Figure 2:**
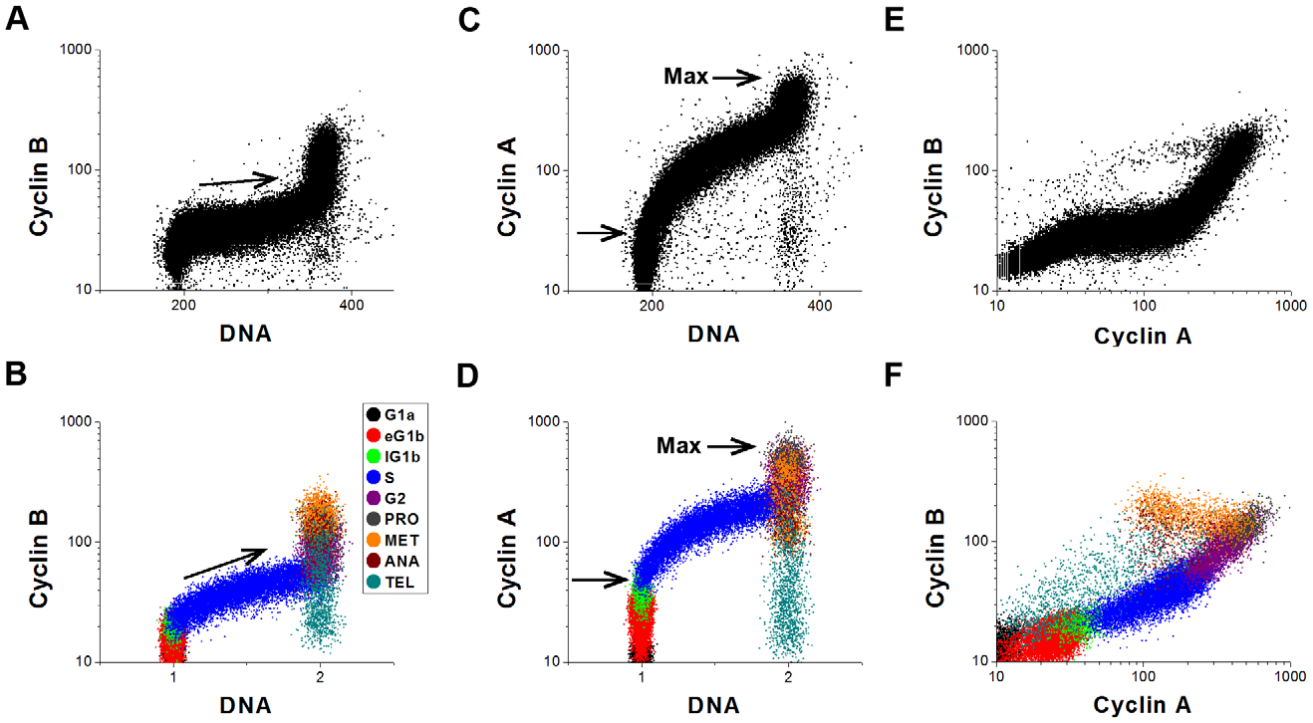
Scatter plots. (A,C,E) Flow cytometry data from Yan et al [42]. DNA = 190 corresponds to G1 and DNA = 380 corresponds to G2/M. (B,D,F) Our simulations. We are plotting the total amount of cyclin A and cyclin B per cell, i.e., [CycA]·*M*(*t*) and [CycB]·*M*(*t*). DNA = 1 in G0/G1 phase;  = 2 in G2/M phase. Some ‘instrumental noise’ has been added to the calculated levels of cyclins and DNA, as described in the Methods. The arrows in (A, B) indicate the rate of cyclin B accumulation in S phase in the measurements and in the model. The arrows in (C, D) indicate the cyclin A level at the onset of DNA synthesis, compared to the maximum expression level of ∼600 AU.

Our first test for the hybrid model is to simulate flow cytometry measurements of the DNA content and cyclin levels in an asynchronous population of RKO (colon carcinoma) cells [42]. In the data set, a typical scatter plot has about 65000 data points, each point displaying the measurements of two observables in a single cell chosen at random from the cell cycle (Fig. 2). When the data are plotted in this way, they form a cloudy tube of points through a projection of the state space (say, cyclin B versus cyclin A). Because there will be some cells from every phase of the cell cycle, the tube closes on itself. If the system were completely deterministic and the measurements were absolutely precise, the data points would be a simple closed curve (a ‘limit cycle’) in the state space. The data actually present a fuzzy trajectory that snakes through state space before closing on itself. The indeterminacy of the points comes (presumably) from two sources: intrinsic noise in the molecular regulatory system (modeled by the random waiting times, 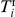) and extrinsic measurement errors, which we shall introduce momentarily. Our strategy for simulating flow-cytometry data is explained in more detail in the Methods section.

**Figure 3:**
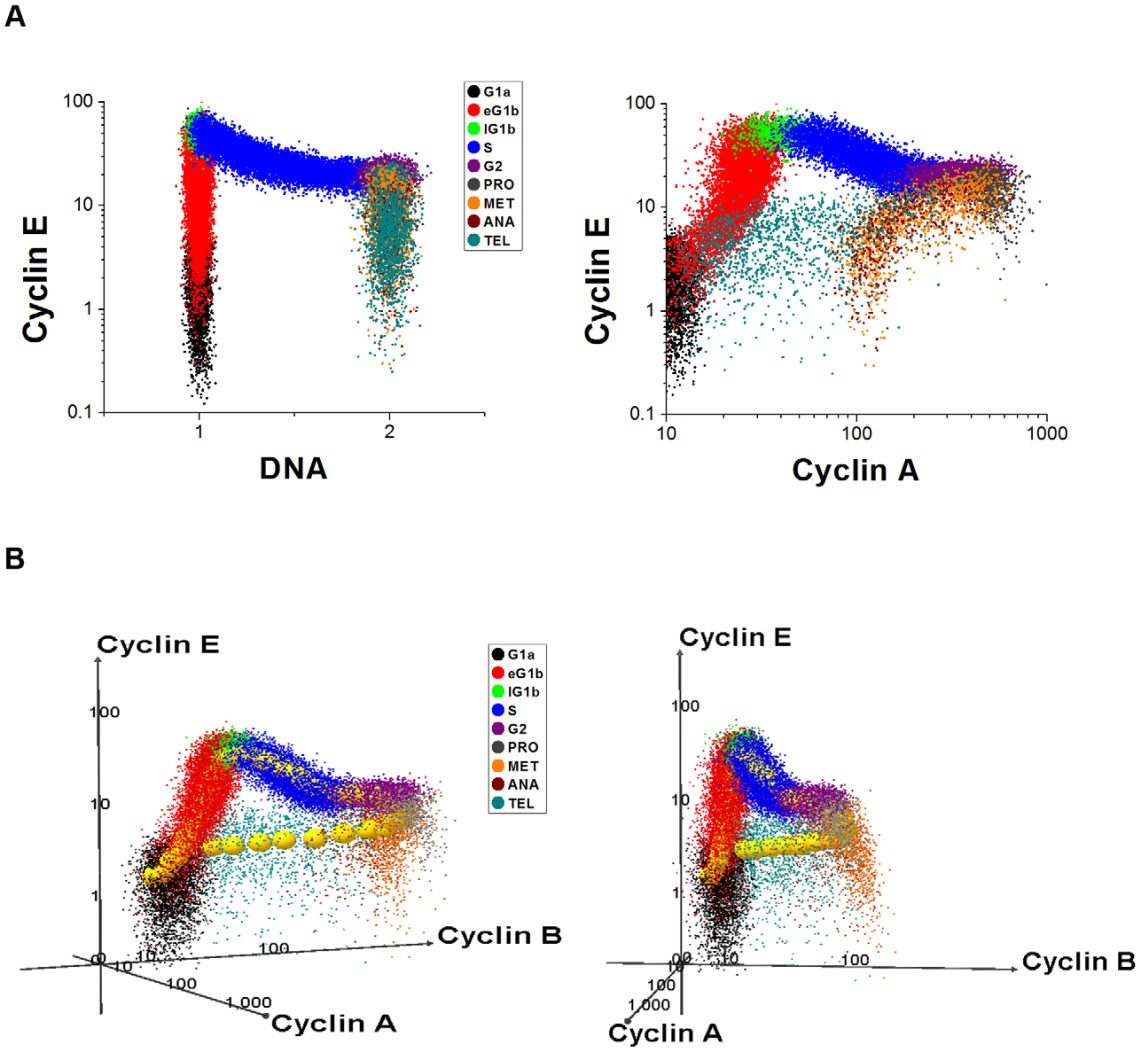
Model predictions of cyclin E dynamics. (A) Scatter plots. (B) Stochastic limit cycle in the state space of cyclins A, B and E. We provide two different perspectives of this three dimensional figure to help visualize how the cyclin levels go up and down. In addition, we have added golden-colored balls to help guide the eye along the cell cycle trajectory. Each ball represents the average of the cyclin levels of all the cells binned over a hundredth of the ϕ*_i_* interval [0,1], where ϕ*_i_* refers to the fraction of the cell cycle completed by cell *i* (as described in the Methods section). Finally, it may help to recognize that Fig. 2E is a projection of the data on the CycA-CycB plane, and Fig. 3B is a projection on the CycA-CycE plane.

In Fig. 2 we compare our simulated flow-cytometry scatter plots with experimental results of Yan et al. [42]. We color-code each cell in the simulated plot according to which Boolean State (Table 1) the cell is in at the time of fixation. In Fig. 3 we plot cyclin E fluctuations, as predicted by our model, along with a projection of the cell cycle trajectory in a subspace spanned by the three cyclin variables (A, B and E).

### Contact inhibition of cultured cells

**Figure 4:**
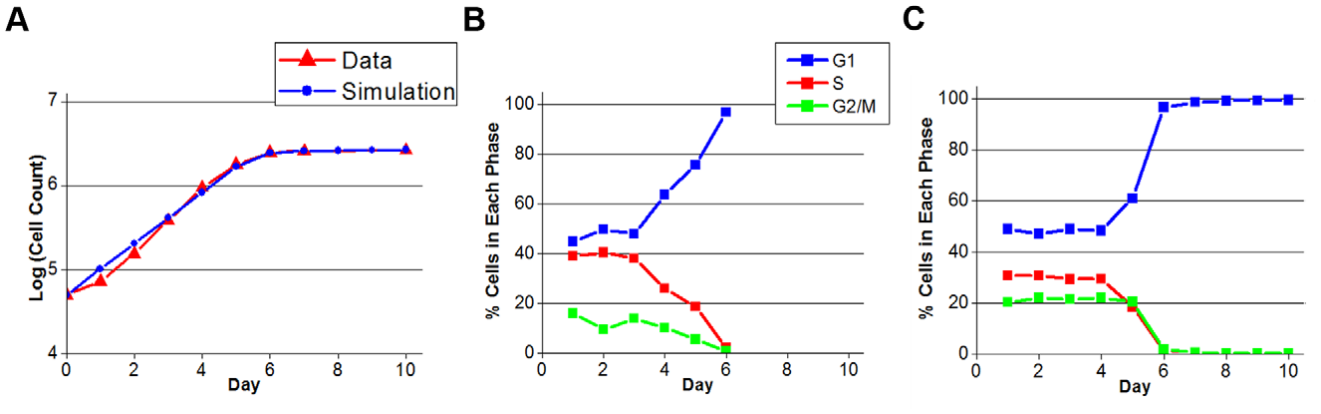
Contact inhibition of a culture of human umbilical vein endothelial cells. (A) Growth curve for the HUVEC population over 10 days, showing the base-10 logarithm of the cell count for both experimental data and our simulation (with *N*_0_ = 11000 and *N*_1_ = 500). (B) Daily distribution of cells across the phases of the cell cycle, from experimental data. (C) Model simulation of the phase distributions.

As a further test of the utility of this modeling approach, we have used our hybrid model to simulate an exponentially growing population of an immortalized Human Umbilical Vein Endothelial cell line (HUVEC). In the experiment (Fig. 4A; see Methods), a culture is seeded with 5×10^4^ cells on ‘Day 0’ and allowed to grow. At Day 6, it reaches confluence and cell number plateaued at a constant level.

To apply the hybrid model to this data, we had to devise a way to model contact inhibition, which arrests cells in a stable quiescent state. To this end, we assume that the transition probability, *p*, for exiting State 1 is a function of the number of cells alive at that time, *N*:
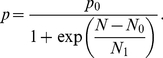
(3)For 0<*N*_1_≪*N*_0_, *p* is a sigmoidal function of *N* that drops abruptly from *p*_0_ to 0 for *N*>*N*_0_. For each cell in this simulation, we set *λ*_1_ (the mean for the random time spent in G1a) to 1/*p*, and we choose *p*_0_ = 0.5 h^−1^ to conform to the value of *λ*_1_ in Table 1. As the population size *N* increases, the time spent in G1a phase increases until cells eventually arrest in State 1, and the growth curve, *N*(*t*), levels off. In this case, State 1 in our model corresponds to a quiescent state (G0) in which cells are alive but not proliferating.

To make the simulation more tractable, we start off with 500 cells (instead of 50,000 cells) and follow the lineage of each initial cell until Day 10. Every 24 hours, we compute the number of cells alive at that point of time and plot the results in Fig. 4A, along with the experimental data (scaled down by a factor of 100). The parameter values, *N*_0_ = 11,000 and *N*_1_ = 500, are chosen to fit the simulation to the observed growth curve. From the model we can also compute the percentage of cells in G0/G1, S and G2/M phases on each day (Fig. 4C), and the results compare favorably with the experimental observations (Fig. 4B). Lastly, we also simulate the patterns of cyclin A2 and cyclin B1 expression on each day for the growing population of HUVEC cells (see Supporting Fig. S1).

## Discussion

We have constructed a simple, effective model of the cyclin-dependent kinase control system in mammalian cells and used the model to simulate faithfully the accumulation and degradation of cyclin proteins during asynchronous proliferation of RKO (colon carcinoma) cells. The model is inspired by the work of Li et al. [14], who proposed a robust Boolean model of cell cycle regulation in budding yeast. Our goal was to retain the elegance of the Boolean representation of the switching network, while introducing continuous variables for cell size, cell age, and cyclin composition, in order to create a model that could be compared in quantitative detail to experimental measurements.

We have shown that this model can accurately simulate flow-cytometric measurements of cyclin abundances in asynchronous populations of growing-dividing mammalian cells. The parameters in the model that allow for a quantitative description of the experimental measurements are easily estimated from the data itself. Now that the model is parameterized and validated for wild-type cells, we are currently extending it to handle the behavior of cell populations perturbed by drugs and by genetic interference. In some cases, only modest extensions of the model are required; in other cases, a more thorough overhaul of the way the discrete and continuous variables interact with each other is necessary.

We have chosen parameter values in our model to capture the major features of cyclin fluctuations as measured by flow cytometry during the somatic division cycle of mammalian cells. We have used a human tumor cell line to calibrate our model. Between cell lines and normal human cultured cells, there are differences in the expressions of A and B cyclins [43]; however, when the levels of cyclin B1 were rigorously compared for HeLa, K562, and RKO cells, both the patterns and magnitudes of expression are remarkably similar, apparently dependent to some degree on the rate of population growth [44]. In addition, the patterns of expression of cyclins A2 and B1 are similar for these human tumor cell lines and stimulated normal human circulating lymphocytes (Supporting Fig. S2). Overall, the simulation outputs have satisfying similarity both in pattern and magnitude to the real data for RKO cells, and our simulated expression patterns of cyclins A, B and E for the tumor cell line are quite similar to the simulated expression patterns in HUVEC cells (see Supporting Fig. S1).

However, there remain some inconsistencies between our mathematical simulations and our experimental observations that point out where future modifications to the model are needed. For example, in the model DNA synthesis starts when cyclin A has accumulated to ∼8% of its maximum level (see arrow in Fig. 2D; 50/600≈8%), whereas in our measurements DNA synthesis starts when cyclin A is ∼5% of its maximum level (arrow in Fig. 2C). This discrepancy is tempered by the fact that we are not confident of the quantitative accuracy of cyclin A expression levels below ∼4% of its maximum level in Fig. 2C. Where we place the minimum expression level of cyclin A in Fig. 2D affects our estimate of the cyclin A level at onset of DNA synthesis (50 AU at present). By lowering the minimum expression level of cyclin A below 10 AU in Fig. 2D (e.g., by lowering *k*′*_sa_*), we could line up the two arrows in Figs. 2C and D. Nonetheless, we observe (Supporting Fig. S3) that cyclin A expression correlates highly with BrdU incorporation, suggesting that significant accumulation of cyclin A begins simultaneously with the onset of DNA synthesis, whereas in our model cyclin A production begins in mid-G1 phase. This discrepancy could be minimized by lowering the cyclin A threshold (*θ_A_*) in the model.

The simulation (Fig. 2B) captures the observed accummulation of cyclin B in late G1 (when Cdh1 turns off), but the simulated rise in cyclin B during S phase appears to be faster than the observed rise [45] (compare the arrows in Figs. 2A and B). The simulation does capture the rapid accumulation of cyclin B observed in G2. Finally, while we did not calibrate the cyclin E expression parameters to any specific dataset, the pattern of expression in Fig. 3A is quite similar to expected expression patterns for normal human somatic cells and some human tumor cell lines [46].

We believe that our hybrid approach will be generally useful for modeling macromolecular regulatory networks in cells, because it combines the qualitative appeal of Boolean models with the quantitative realism of reaction kinetic models.

## Methods

### Simulations

We simulate a flow cytometry experiment with our hybrid model in two steps.

*Step 1: Creating complete ‘life histories’ for thousands of cells.* At the start of the simulation, we specify initial conditions at the beginning of the cycle (State 1) for a progenitor cell. We used the following initial values of the state variables: [CycA] = [CycB] = [CycE] = 1 and *M* = 3. Our strategy is to follow this cell through its cycle until it divides into two daughters. We then choose one of the two daughters at random and repeat the process, continuing for 32500 iterations. We discard the first 500 cells, and keep a sample of 32000 cells that have completed a replication-division cycle according to our model. In the second step, we create a simulated sample of 32000 cells chosen at random phases of the cell cycle, to represent the cells that were assayed by the flow cytometer.

Let us consider cell *i* (1<*i*<32500) at the time of its birth, *t_i_*_0_. By definition, this cell is in State 1, and we assume that we know its birth mass, *M*(*t_i_*_0_), and its starting concentrations of cyclins A, B and E. Denote the starting concentrations as [CycA(*t_i_*_0_)], [CycB(*t_i_*_0_)], [CycE(*t_i_*_0_)]. In the ensuing discussion, unless it is necessary for clarity, we drop the *i* subscript, it being understood that we are talking about a representative cell in the population. We will follow this cell until it divides to produce a daughter cell with known concentrations of cyclins.

According to Table 1, a cell in State 1 has no special conditions to satisfy before moving to State 2. Hence the residence time in State 1 is a random number 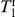 chosen from an exponential distribution with mean *λ*_1_ = 2 h. The cell enters State 2 at *t*_1_ = *t*_0_+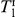. Assuming exponential growth, its size at this time is *M*(*t*_1_) = *M*(*t*_0_) exp{γ(*t*_1_−*t*_0_)} = *M*(*t*_0_) exp{γ*A*_1_}, where γ is the specific growth rate of the culture and *A*_1_ = *t*_1_−*t*_0_ is the age of the cell when it exits State 1. To illustrate how cyclin concentrations are computed at *t* = *t*_1_, let us consider cyclin A as an example. During the interval *t*_0_<*t*<*t*_1_, [CycA] satisfies a linear ODE with effective rate constants *k*_sa1_ = *k*′_sa_ = 5 and *k*_da1_ = *k*′_da_+*k*″′_da_ = 1.4, because *B*_TFE_ = *B*_TFB_ = *B*_Cdc20A_ = 0 and *B*_Cdh1_ = 1 for a cell in State 1. We can compute the concentration of cyclin A at any time during this interval from
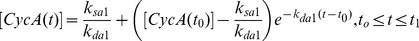
(4)Setting *t* = *t*_1_ in this equation gives the number we seek. In this fashion, we start tabulating the following information for each simulated cell:
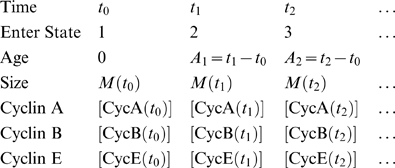



Notice that, at *t* = *t*_1_ when the cell enters State 2, the transcription factor (TFE) for cyclins E and A turns on, and these cyclins start to accumulate. The cell cannot leave State 2 until cyclin E accumulates to a sufficiently high level: [CycE](*t*)·*M*(*t*) = *θ*_E_, according to Table 1. When this condition is satisfied, the cell leaves State 2 and enters State 3. The size dependence on this transition is a way to couple cell growth to the DNA replication-division cycle. According to the parameter settings in Table 1, there is no stochastic component to the transition out of State 2.

We continue in this fashion until the cell leaves State 9 and returns to State 1, when cyclin B is degraded at the end of mitosis. This is the signal for cell division. The age of the cell at division is *A*_9_ = *t*_9_−*t*_0_, and the mass of the cell at division is *M*(*t*_9_) = *M*(*t*_0_) exp(γ·*A*_9_). The mass of the daughter cell at the beginning of her life history is *M*_daughter_(*t*_0_) = *δ*·*M*_mother_(*t*_9_), where *δ* is a random number sampled from a normal distribution of mean 0.5 and standard deviation 0.0167 to allow for asymmetries of cell division.

Notice that simulating the life history of a single cell only requires generating about a dozen random numbers and performing a handful of algebraic calculations. At no point do we need to solve differential equations numerically. Hence we can quickly calculate the life histories of tens of thousands of cells.

*Step 2: Finding the DNA and cyclin levels of each cell in an asynchronous sample*. In the flow cytometry experiments of Yan et al. [42], a random sample of cells is taken from an asynchronous population, the cells are fixed and stained, and then run one-by-one through laser beams where fluorescence measurements are made. So each data point consists of measurements of light scatter (related to cell size) and fluorescence proportional to DNA and cyclin content for a single cell taken at some random point in the cell cycle. To simulate this experiment we must assign to each of our 32000 simulated cells a number *ϕ_i_* selected randomly from the interval [0,1], where *ϕ_i_* refers to the fraction of the cell cycle completed by cell *i* when it was fixed and stained for measurement. Because each mother cell divides into two daughter cells, the density of cells at birth, *ϕ* = 0, is twice the density of cells at division, *ϕ* = 1. The ‘ideal’ probability density for an asynchronous population of cells expanding exponentially in number is
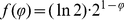
(5)According to the ‘transformation method’ [47, Chapter 7.2], we compute *ϕ* as
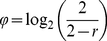
(6)where *r* is a random number chosen from a uniform distribution on [0,1]. In this way, we generate 32000 fractions, *ϕ_i_*.

If *ϕ_i_* is the cell-cycle location of the *i* ^th^ cell when it is selected for the flow cytometry measurements, then its age at the time of selection is *a_i_* = *ϕ_i_*·*A_i_*_9_, where *A_i_*_9_ is the age of the *i* ^th^ cell at division. Given a value for *a_i_*, we then find the state *n* ( = 1, 2, … or 9) of the *i* ^th^ cell at the time of its selection:
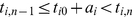
(7)where *t_i_*_,*n*_ (as defined above) is the time at which the *i* ^th^ cell left state *n* to enter state *n*+1.

Once we know the state *n* of the cell, we can compute the concentration of each cyclin in the cell at its exact age *a_i_* by analogy to Eq. [4]:
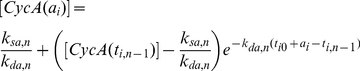
(8)where *k*_sa,*n*_ and *k*_da,*n*_ are the synthesis and degradation rate constants for cyclin A in state *n*. This is a straightforward calculation because in Step 1 we stored the values of *t_n_* and [CycA(*t_n_*)] for every state of each cell. We can also calculate the mass of cell *i* at the time of its selection:
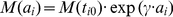
(9)where *M*(*t_i_*_0_) is the mass at birth of cell *i* and γ is the specific growth rate of the culture. Because the flow cytometer measures the total amount of fluorescence proportional to all cyclin A molecules in the *i* ^th^ cell, we take as our measurable the product of [CycA(*a_i_*)] times *M*(*a_i_*).

Lastly we determine the DNA content of cell *i* at age *a_i_* according to:

DNA = 1 for *t_i_*_0_≤*t_i_*_0_+*a_i_*<*t_i_*_3_ = entry of *i* ^th^ cell into S phase
DNA = 1+(*t_i_*_0_+*a_i_*−*t_i_*_3_)/(*t_i_*_4_−*t_i_*_3_) for *t_i_*_3_≤*t_i_*_0_+*a_i_*<*t_i_*_4_ = exit of *i* ^th^ cell from S phase
DNA = 2 for *t_i_*_4_≤*t_i_*_0_+*a_i_*<*t_i_*_9_

Now we have simulated values for the measurable quantities of each cell at the time point in the cell cycle when it was selected for analysis. Before plotting these numbers, we should take into account experimental errors, such as probe quality, fixation, staining and measurement. We do so by multiplying each measurable quantity (DNA content and cyclin levels) by a random number chosen from a Gaussian distribution with mean 1 and standard deviation = 0.03 for DNA measurements and 0.15 for cyclin measurements. These choices give scatter to the simulated data that is comparable to the scatter in the experimental data.

Source codes for the hybrid model are provided in the Supporting Text S1.

### Cells, culture, and fixation

Culture and fixation of RKO cells were described in [42]. The immortalized HUVEC cells [48] at passage 93 were seeded at 2.5×10^3^ cells/cm^2^ in 10 ml EGM-2 media with 2% fetal bovine serum (Lonza, Basel). Duplicate plates were prepared for each time point at days 1, 2, 3, 4, 5, 6, 7, 10, and 15. Cells were fed every other day by replacing half the volume of used media. At the indicated times, cells were trypsinized, washed, and cell counts performed with a Guava Personal Cytometer (Millipore, Billerica, MA). Fixation was as previously described [49]; briefly, cells were treated with 0.125% formaldehyde (Polysciences, Warrington, PA) for 10 min at 37°C, washed, then dehydrated with 90% Methanol. Cells were fixed in aliquots of 1×10^6^ cells (days 1–3) or 2×10^6^ (days 4–15). Fixed cell samples were stored at −20°C until staining for cytometry.

### Immunofluorescence staining, antibodies, flow cytometry

Staining and cytometry for RKO cells were described in [42]. Briefly, cells were trypsinized, fixed with 90% MeOH, washed with phosphate buffered saline, then stained with monoclonal antibodies reactive with cyclin B1, cyclin A, phospho-S10-histone H3, and with 4′,6-diamidino-2-phenylindole (DAPI). For a detailed, updated version of antibodies, staining, and cytometry for cyclins A2 and B1, phospho-S10-histone H3, and DNA content, see Jacobberger et al. (38).

### Data pre-processing

Data pre-processing was performed with WinList (Verity Software House, Topsham, ME). Doublet discrimination (peak versus area DAPI plot) was used to limit the analysis to singlet cells; non-specific binding was used to remove background fluorescence from the total fluorescence related to cyclin A2 and B1 staining. The phycoerythrin channel (cyclin A2) was compensated for spectral overlap from FITC or Alexa Fluor 488. For simplification, very large 2C G1 HUVEC cells and any cells cycling at 4C→8C were removed from the analysis. These were present at low frequency. Data were written as text files then transferred to Microsoft Excel.

## Supporting Information

Figure S1 Patterns of Cyclin A and Cyclin B expression in simulated populations of HUVECs growing toward confluence over days 0–10.
(0.75 MB PDF)

Figure S2 Pattern of Cyclin A expression in stimulated human circulating lymphocytes.
(0.05 MB PDF)

Figure S3 Correlation of Cyclin A expression with BrdU labeling.
(0.33 MB PDF)

Text S1 Source codes.
(0.18 MB DOC)
