# Supplementary material for: A Hybrid Model of Mammalian Cell Cycle Regulation
Source: PLoS Comput Biol. 2011 Feb 10;7(2):e1001077. doi: 10.1371/journal.pcbi.1001077 (PMC3037389; doi:10.1371/journal.pcbi.1001077)
Supplement: Figure S1 — Patterns of Cyclin A and Cyclin B expression in simulated populations of HUVECs growing toward confluence over days 0–10. (0.75 MB PDF) [file pcbi.1001077.s001.pdf]

Figure S1: Patterns of Cyclin A and Cyclin B expression in simulated populations of HUVECs growing toward confluence over days 0—10. More details of the simulations reported in Figure 4 are given in these three sets of scatter plots.

- Set 1: Cyclin A versus DNA.
- Set 2: Cyclin B versus DNA.
- Set 3: Cyclin A versus Cyclin B.

HUVEC Set 1: Cyclin A vs DNA simulations Days 0-10

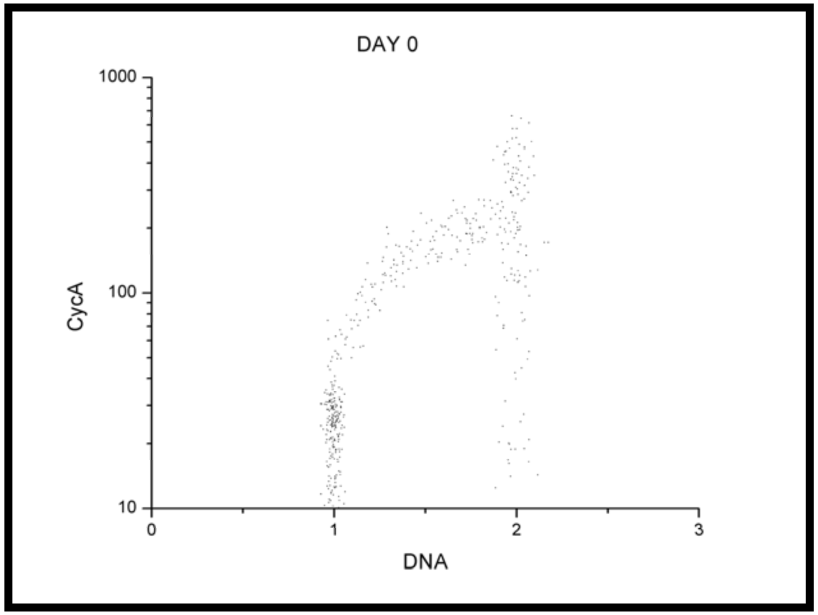

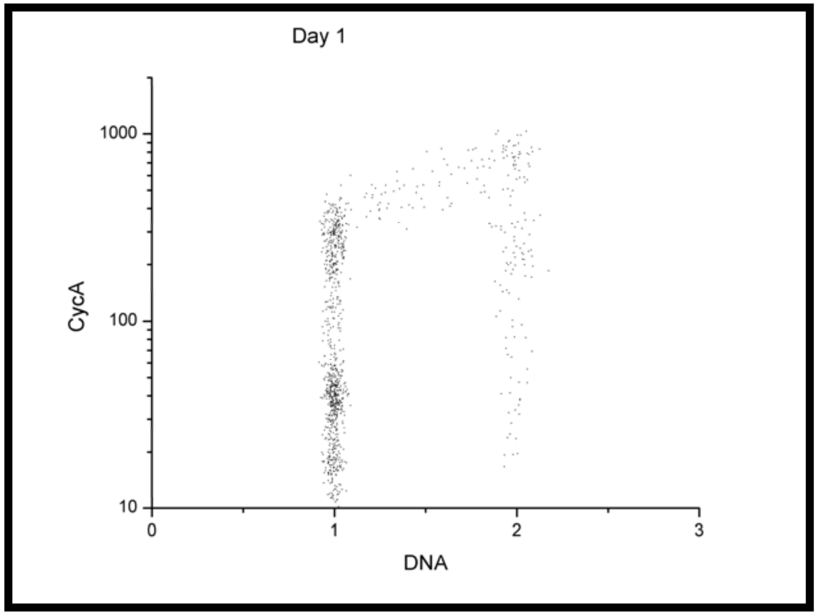

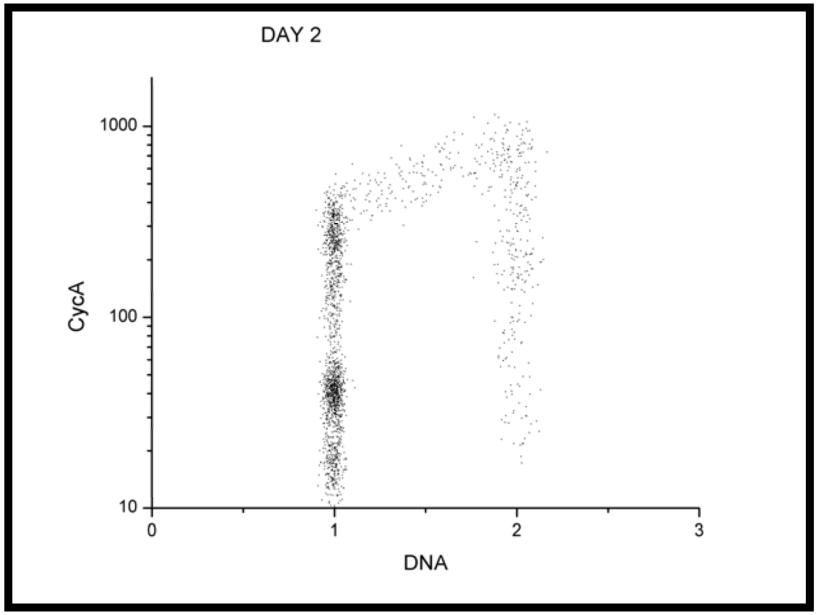

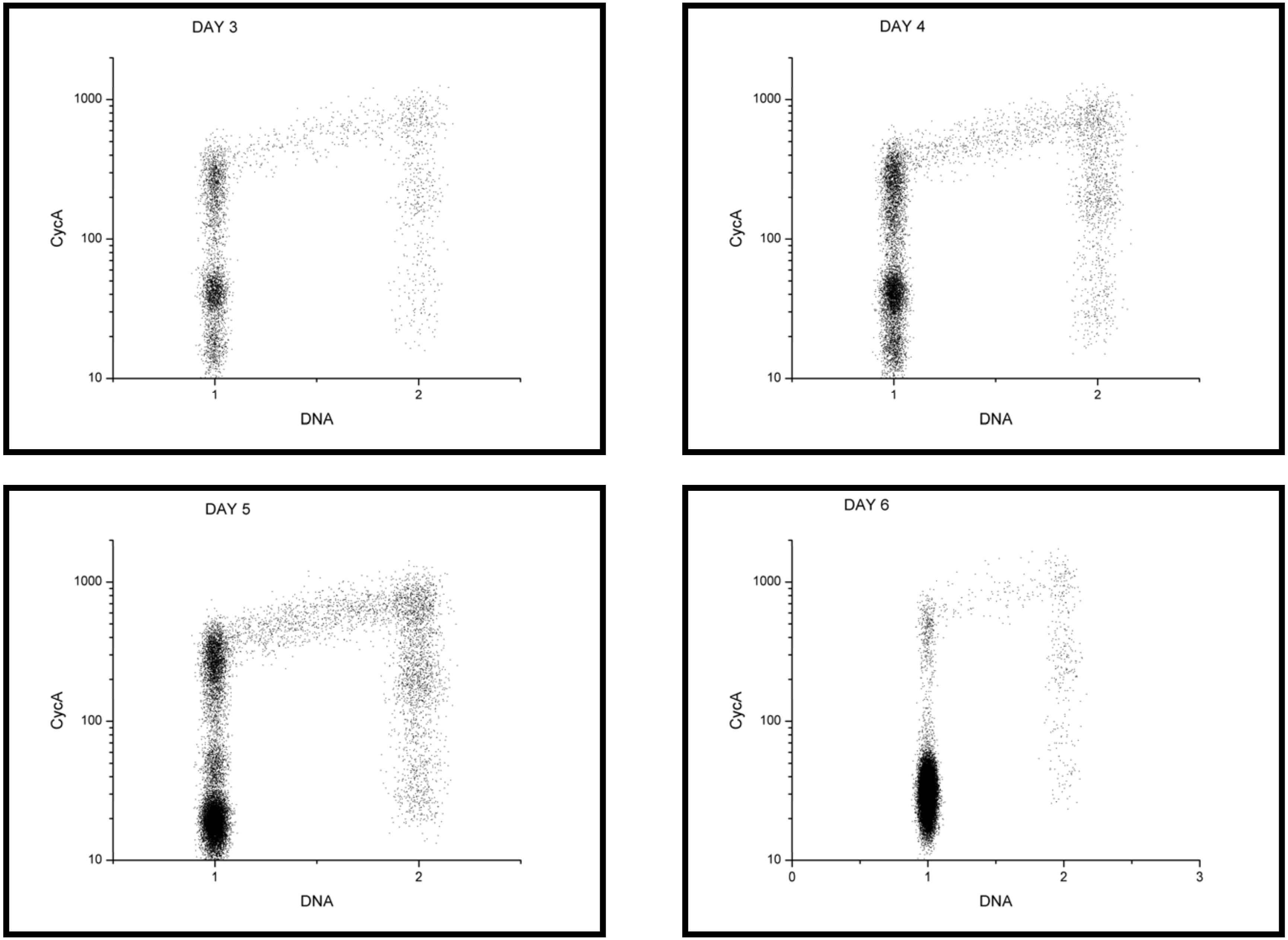
DAY 3
CycA
1000
100
10
1
2
DNA
DAY 4
CycA
1000
100
10
1
2
DNA
DAY 5
CycA
1000
100
10
1
2
DNA
DAY 6
CycA
1000
100
10
0
1
2
3
DNA

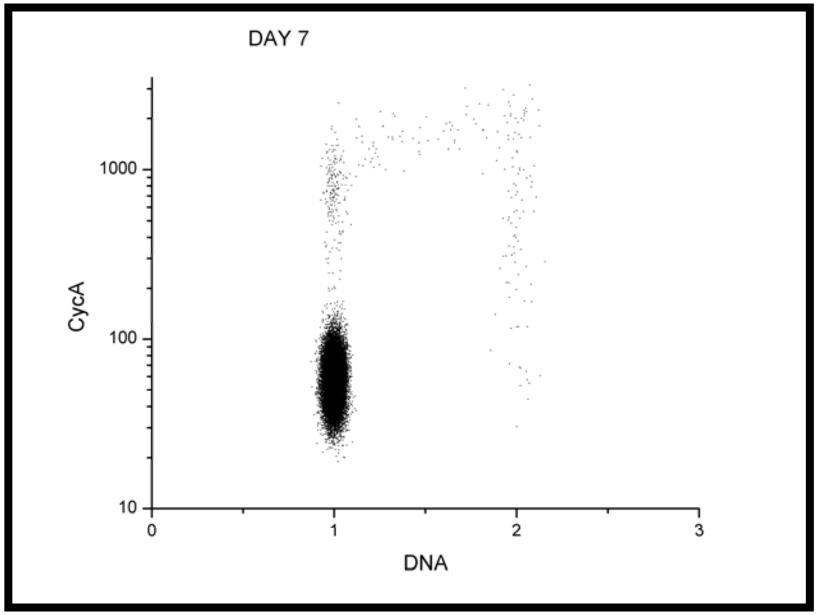
DAY 7
CycA
1000
100
10
0
1
2
3
DNA

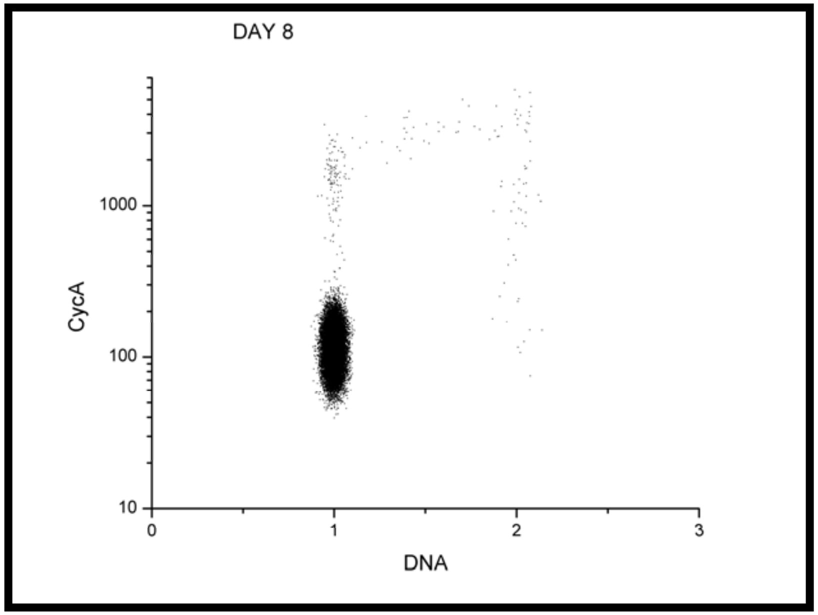
DAY 8
CycA
1000
100
10
0
1
2
3
DNA

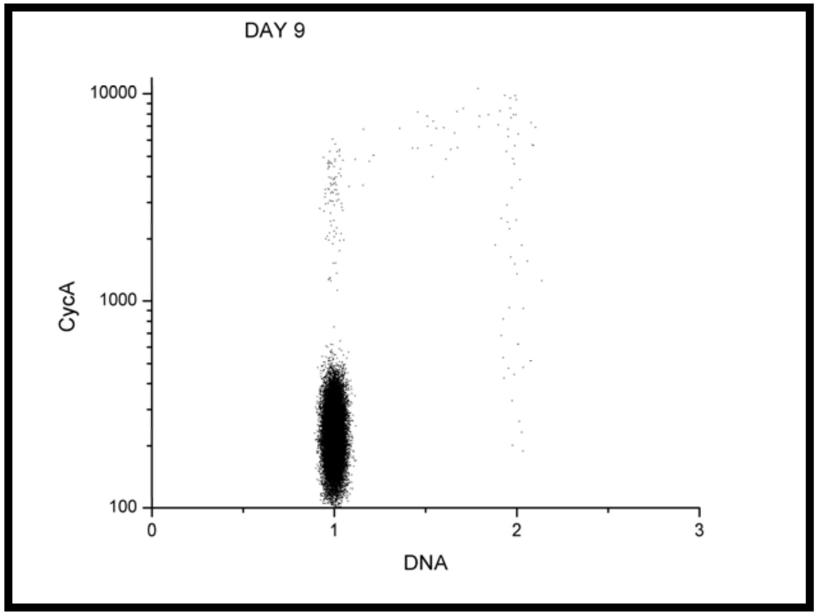
DAY 9
CycA
10000
1000
100
0
1
2
3
DNA

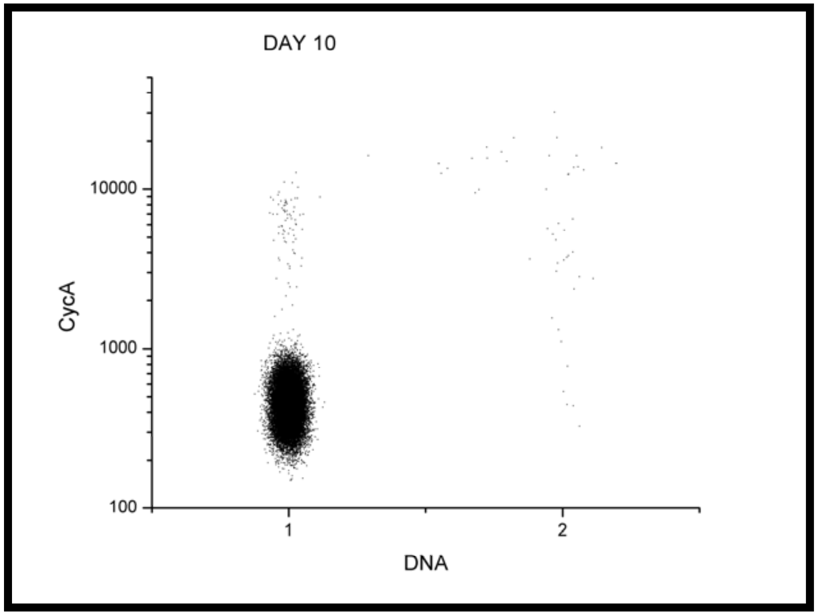
DAY 10
CycA
10000
1000
100
1
2
DNA

HUVEC Set 2: Cyclin B vs DNA simulations Days 0-10

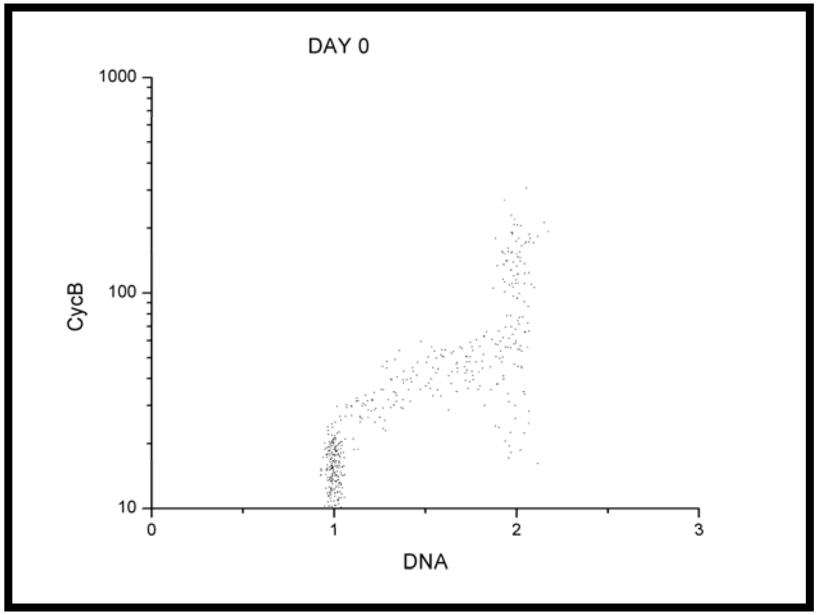

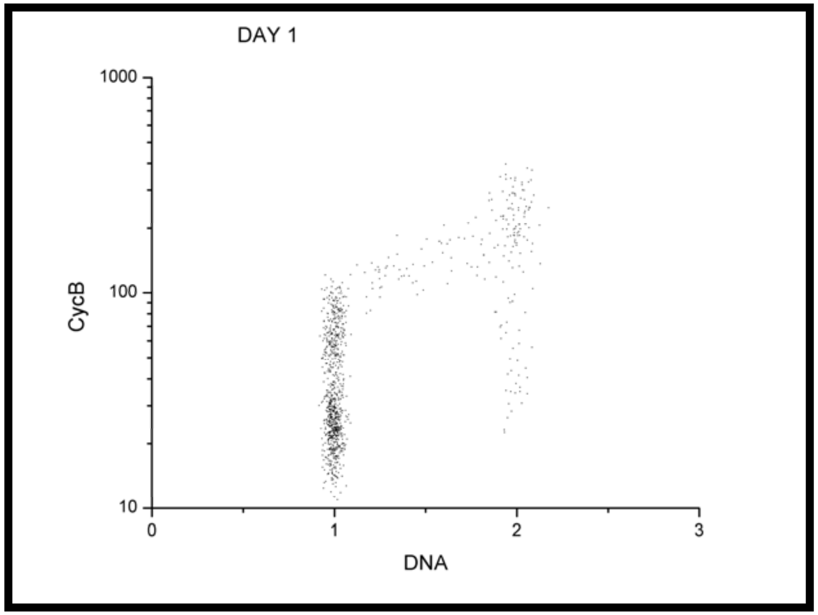

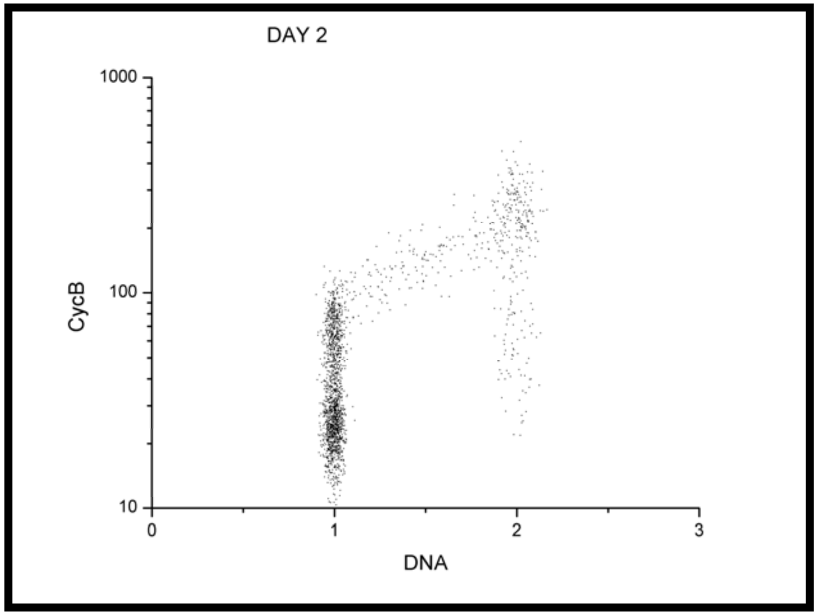

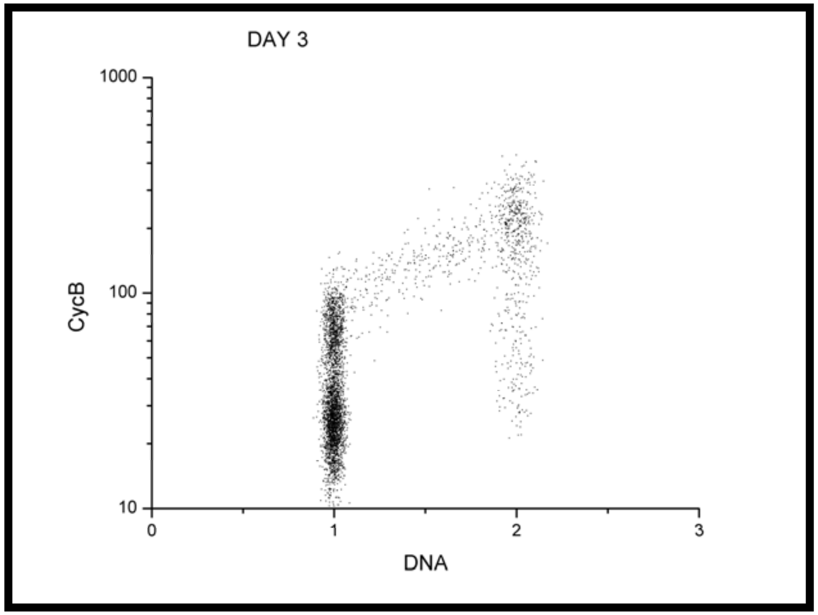
DAY 3
CycB
1000
100
10
0
1
2
3
DNA

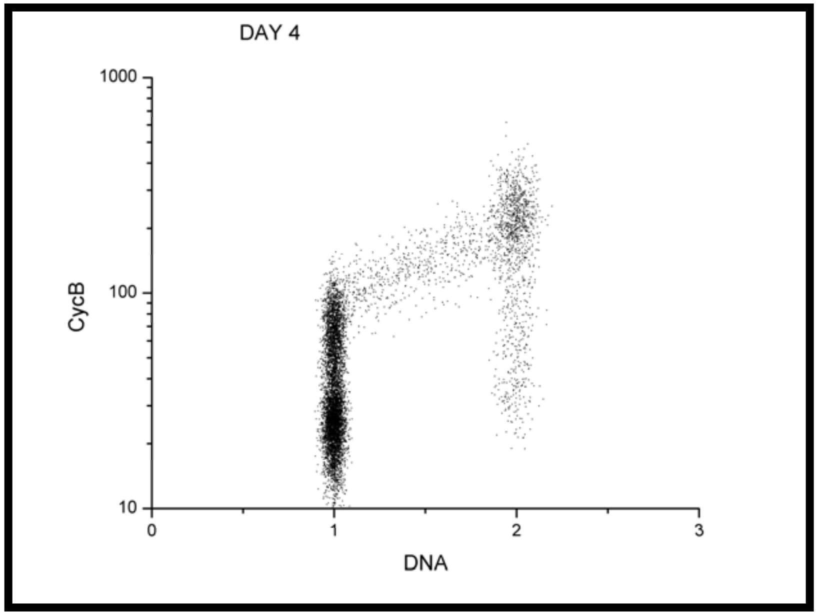
DAY 4
CycB
1000
100
10
0
1
2
3
DNA

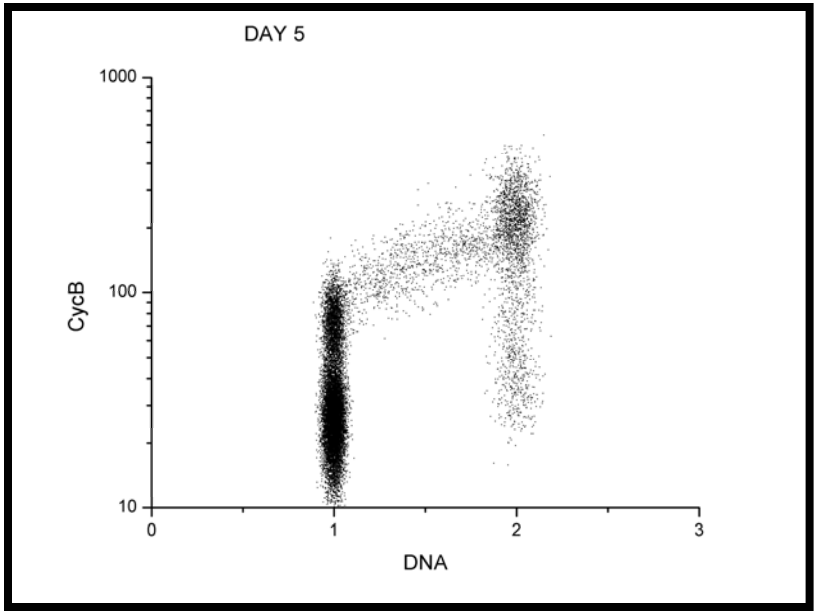
DAY 5
CycB
1000
100
10
0
1
2
3
DNA

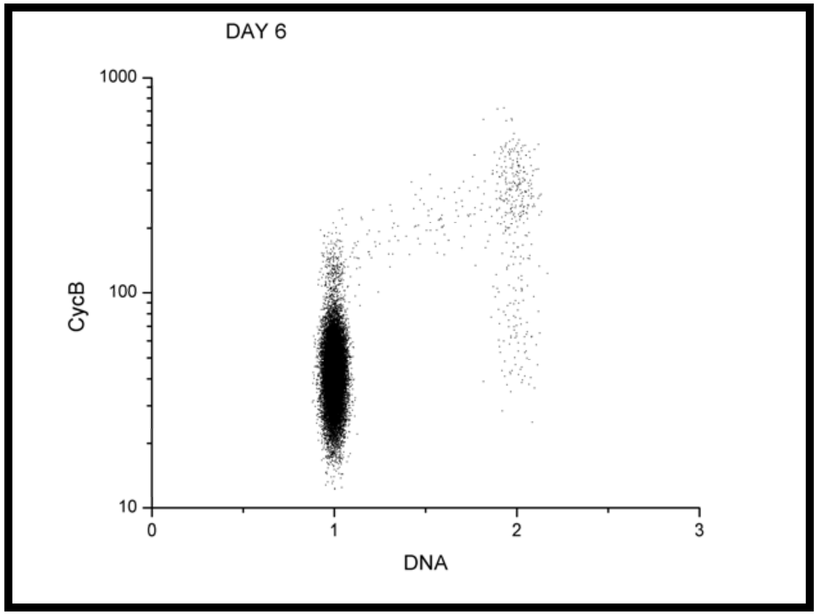
DAY 6
CycB
1000
100
10
0
1
2
3
DNA

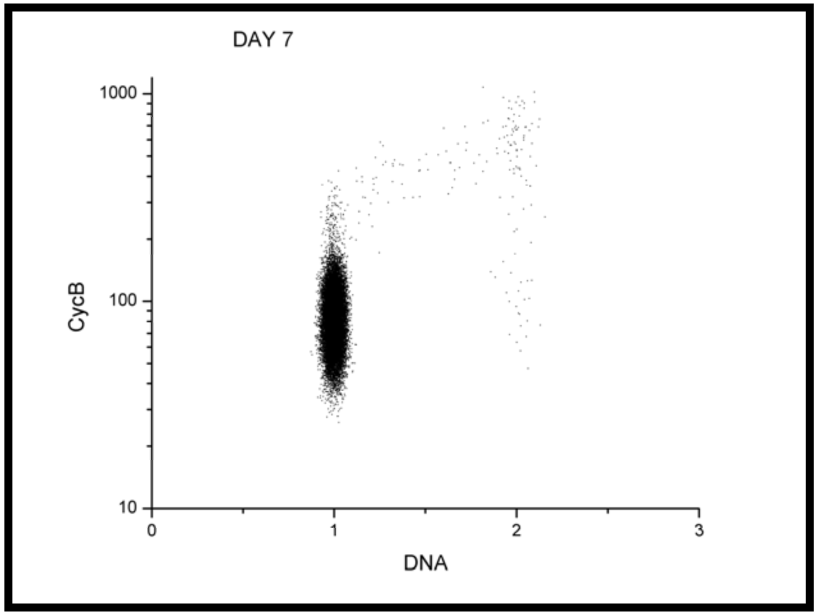
DAY 7
CycB
1000
100
10
0
1
2
3
DNA

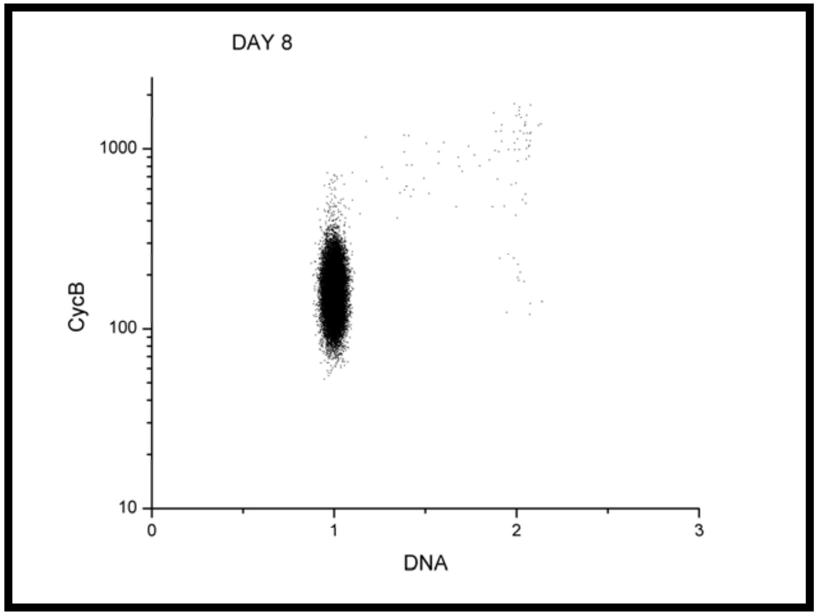
DAY 8
CycB
1000
100
10
0
1
2
3
DNA

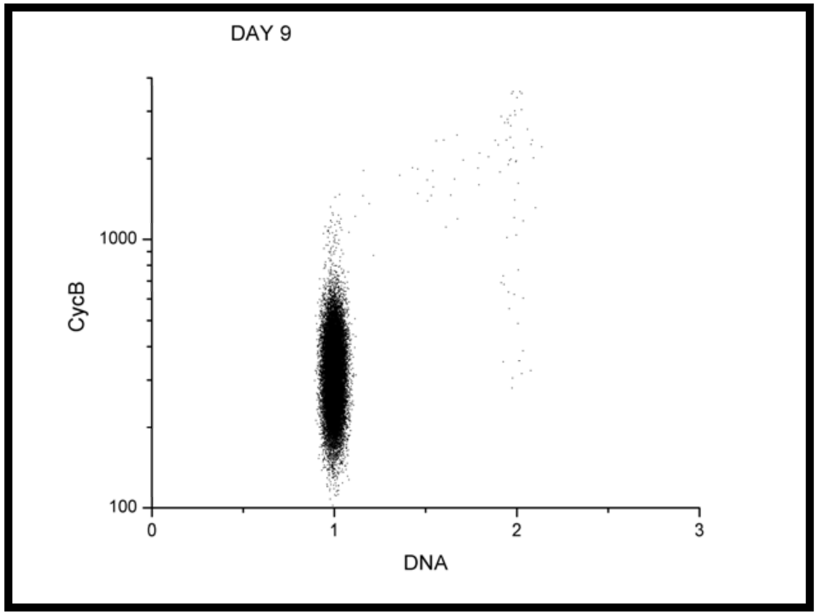
DAY 9
CycB
1000
100
0
1
2
3
DNA

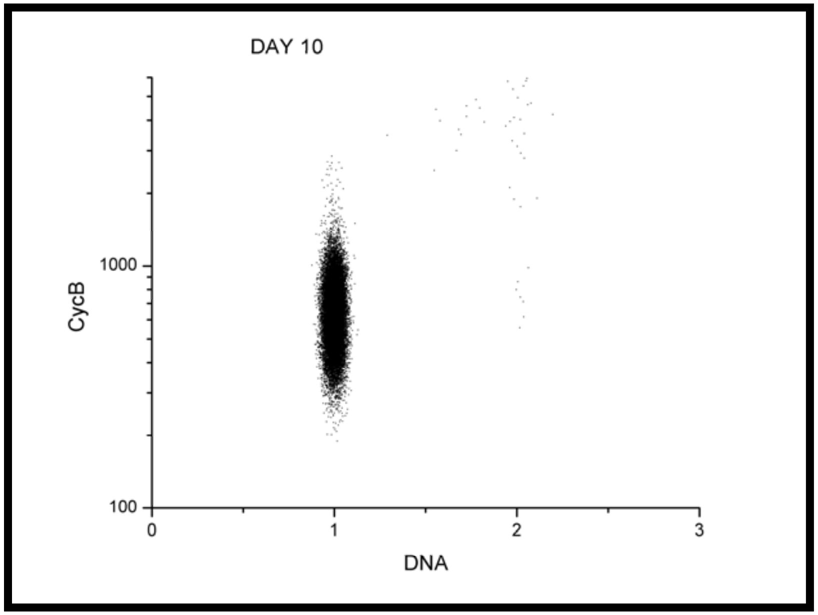
DAY 10
CycB
1000
100
0
1
2
3
DNA

HUVEC Set 3:
Cyclin B vs Cyclin A simulations
Days 0-10

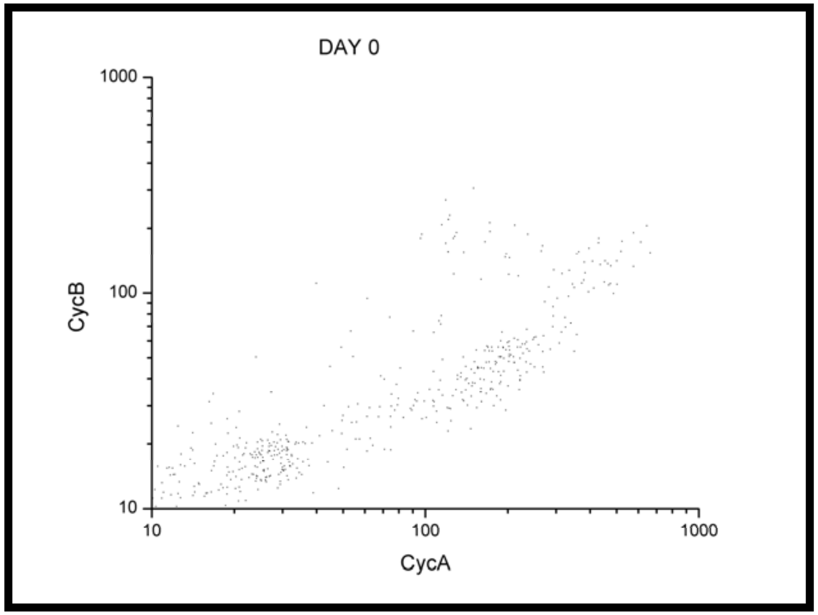

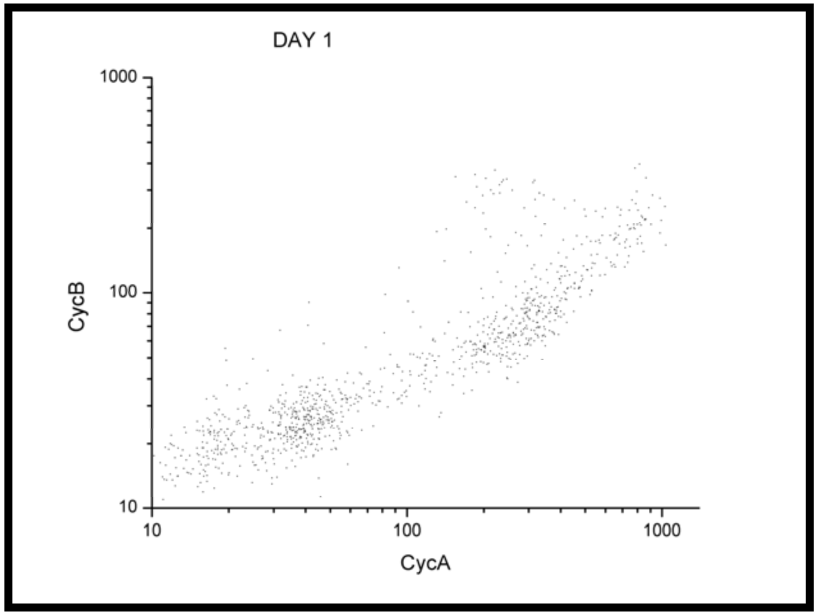

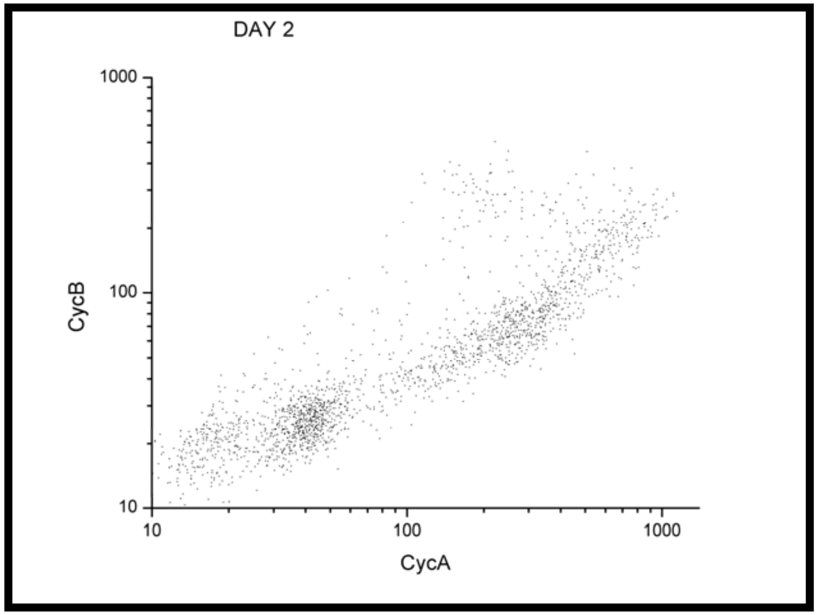

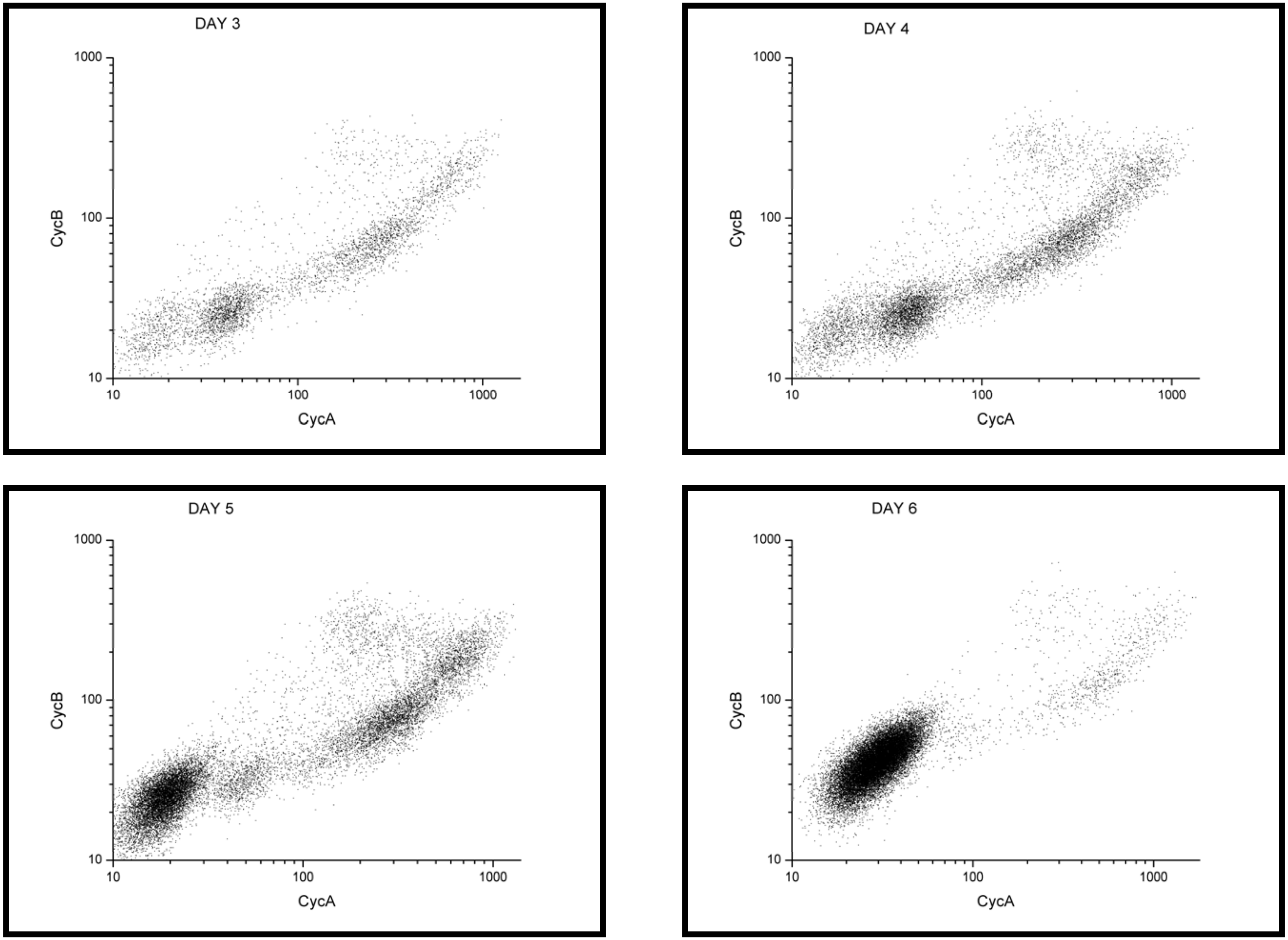

DAY 3
CycB
CycA
DAY 4
CycB
CycA
DAY 5
CycB
CycA
DAY 6
CycB
CycA

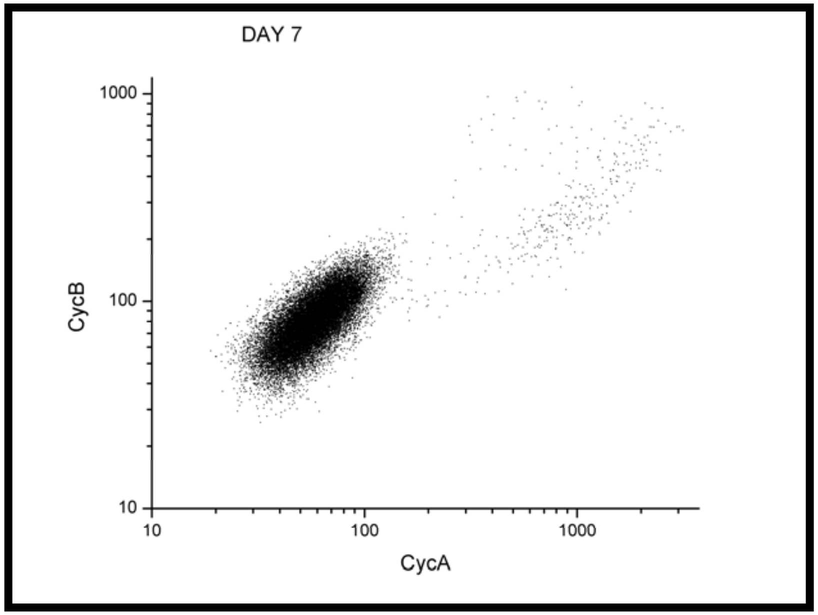
DAY 7
CycB
CycA
1000
100
10
10
100
1000

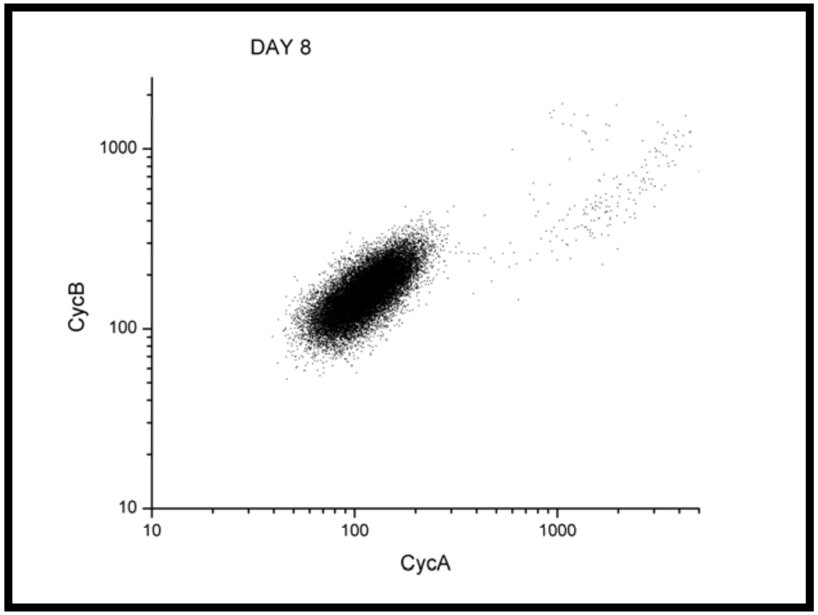
DAY 8
CycB
CycA
1000
100
10
10
100
1000

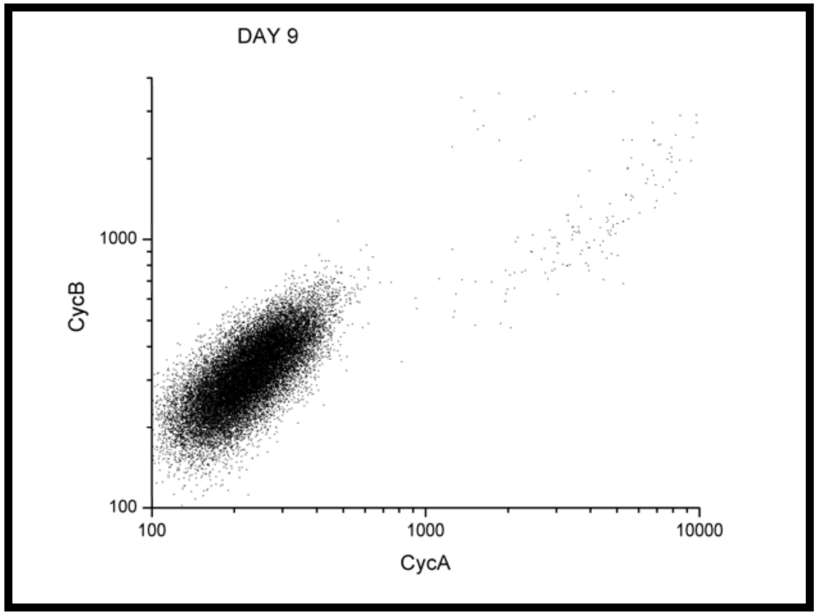
DAY 9
CycB
CycA
1000
100
100
1000
10000

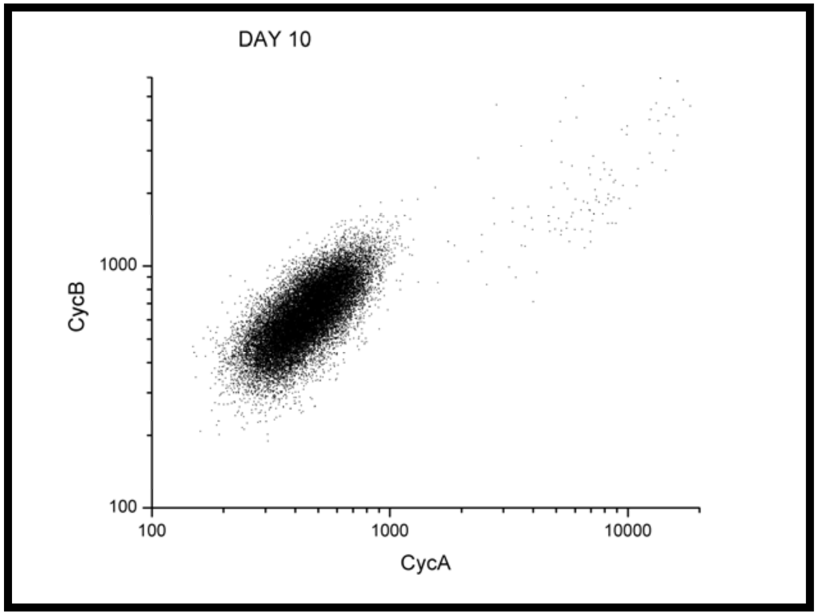
DAY 10
CycB
CycA
1000
100
100
1000
10000
