# Supplementary material for: A Hybrid Model of Mammalian Cell Cycle Regulation
Source: PLoS Comput Biol. 2011 Feb 10;7(2):e1001077. doi: 10.1371/journal.pcbi.1001077 (PMC3037389; doi:10.1371/journal.pcbi.1001077)
Supplement: Figure S2 — Pattern of Cyclin A expression in stimulated human circulating lymphocytes. (0.05 MB PDF) [file pcbi.1001077.s002.pdf]

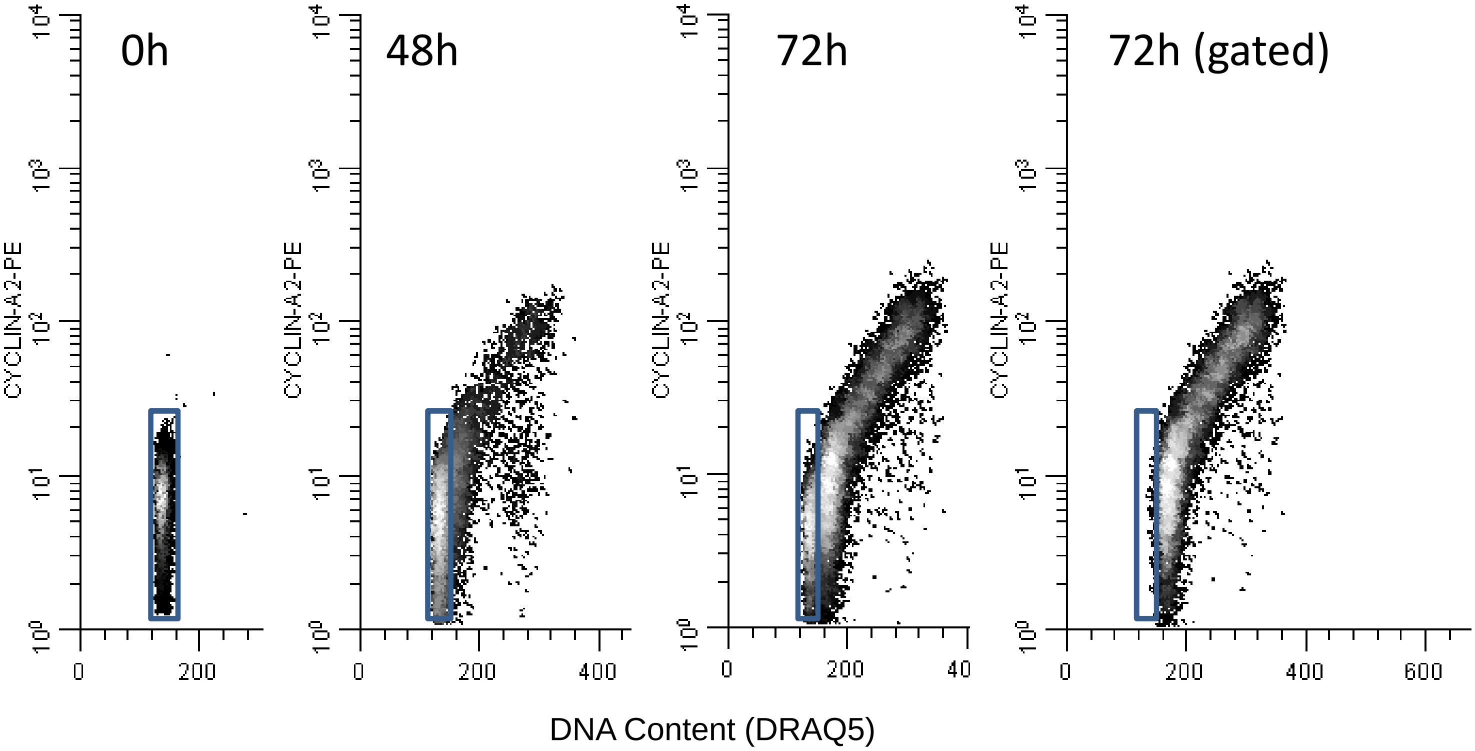

Figure S2: Pattern of Cyclin A expression in stimulated human circulating lymphocytes. Peripheral blood monocytic cells were isolated by Ficoll-Hypaque centrifugation then stimulated in culture with phytohemagglutinin in RPMI-1640 media with 10% fetal bovine serum. At timed intervals, samples were fixed with methanol, then immunofluorescently stained for cyclin A2, MPM-2, and DNA content with DRAQ5. Cycling lymphocytes stain slightly stronger with DRAQ5 than resting lymphocytes, due to size-related non-DNA dye binding. Resting lymphocytes (G0 cells) are demarcated by the blue boxes. The 72h (gated) plot has been gated for MPM-2 positivity, which cleanly separates resting from activated lymphocytes. Cyclin A2 abundance is plotted versus DNA content. At 0 h, all cells are quiescent. By 48 h, many cells have entered the cell cycle and show a pattern of Cyclin A expression that is similar to the cell lines analyzed in the text. By MPM-2 staining and DNA content, we estimate that only 19% of cells remain in G0 at 72 h.
