# Supplementary material for: A Hybrid Model of Mammalian Cell Cycle Regulation
Source: PLoS Comput Biol. 2011 Feb 10;7(2):e1001077. doi: 10.1371/journal.pcbi.1001077 (PMC3037389; doi:10.1371/journal.pcbi.1001077)
Supplement: Figure S3 — Correlation of Cyclin A expression with BrdU labeling. (0.33 MB PDF) [file pcbi.1001077.s003.pdf]

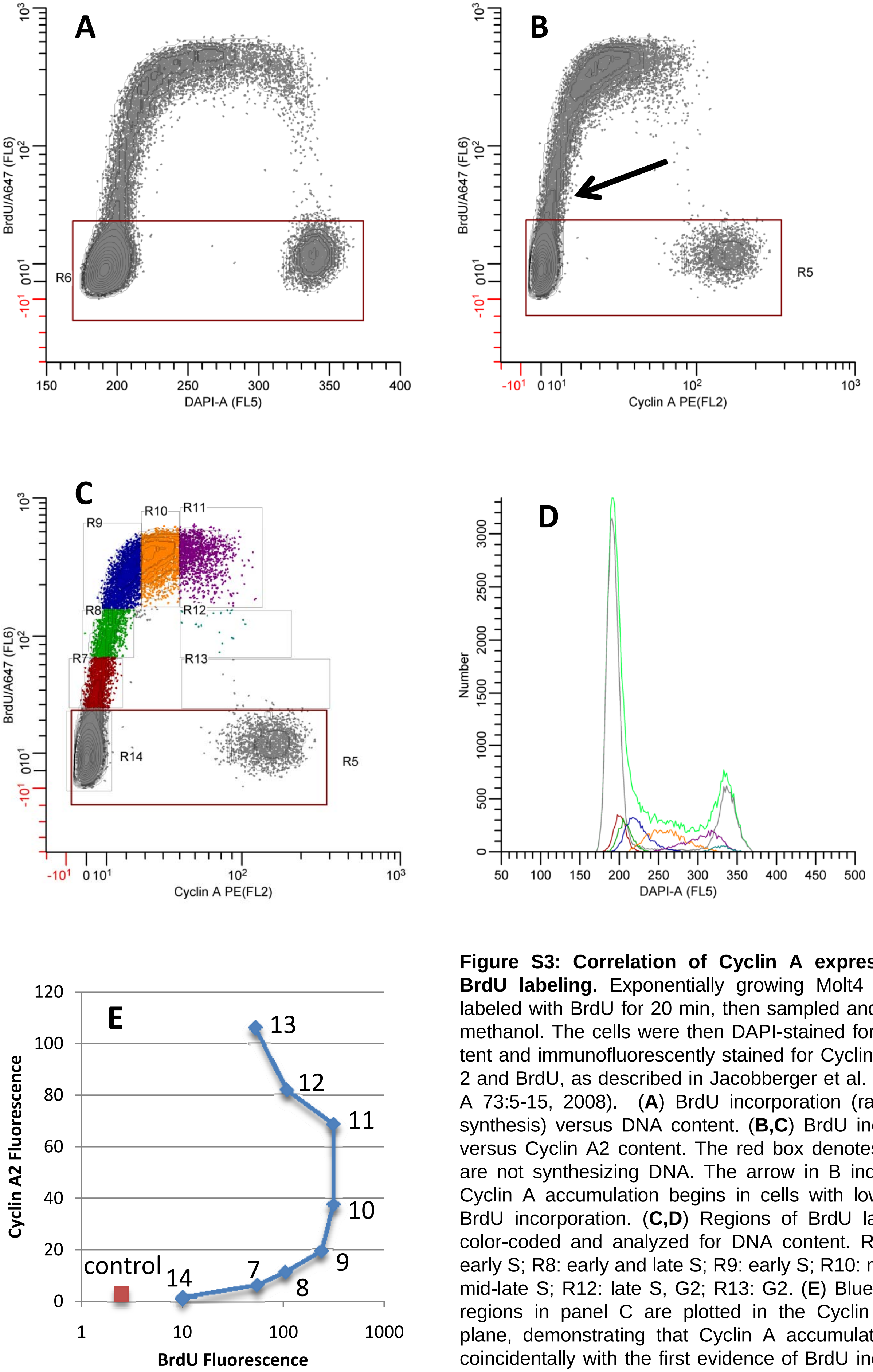

Figure S3: Correlation of Cyclin A expression with BrdU labeling. Exponentially growing Molt4 cells were labeled with BrdU for 20 min, then sampled and fixed with methanol. The cells were then DAPI-stained for DNA content and immunofluorescently stained for Cyclin A2, MPM-2 and BrdU, as described in Jacobberger et al. (Cytometry A 73:5-15, 2008). (**A**) BrdU incorporation (rate of DNA synthesis) versus DNA content. (**B,C**) BrdU incorporation versus Cyclin A2 content. The red box denotes cells that are not synthesizing DNA. The arrow in B indicates that Cyclin A accumulation begins in cells with low levels of BrdU incorporation. (**C,D**) Regions of BrdU labeling are color-coded and analyzed for DNA content. R7: G1 and early S; R8: early and late S; R9: early S; R10: mid S; R11: mid-late S; R12: late S, G2; R13: G2. (**E**) Blue curve: the regions in panel C are plotted in the Cyclin A2—BrdU plane, demonstrating that Cyclin A accumulation begins coincidentally with the first evidence of BrdU incorporation in early S phase (Region 7). Red square: negative control (no BrdU label).
