# Supplementary material for: A Hybrid Model of Mammalian Cell Cycle Regulation
Source: PLoS Comput Biol. 2011 Feb 10;7(2):e1001077. doi: 10.1371/journal.pcbi.1001077 (PMC3037389; doi:10.1371/journal.pcbi.1001077)
Supplement: Text S1 — Source codes. (0.18 MB DOC) [file pcbi.1001077.s004.doc]

**Supporting Information Text S1**

**Source Code 1: Implementing the hybrid model used to generate the simulation plots in Figures 2 & 3 (Pages 2—28).** The hybrid model described in the paper is implemented in C++, using Microsoft Visual Studio 2008 Express Edition. The output is recorded in text files with the appropriate tabular format; the 2D plots are made using OriginPro8, and the 3D plots using XLSTAT, an Excel plug-in. The first section of the implementation sets up all the model parameters, including the kinetic rate constants. Then there is a major *for* loop which iterates through 32,500 successive cell cycles to create our population of 32,000 cells (the first 500 are discarded to get rid of initial condition effects). For each cell, the deterministic and random time spent in each state is computed, and the cyclin concentrations at the end of each state are also computed, by calling a function which contains the Boolean values for a given cyclin’s regulators in all the states. The total time spent in each state and the cyclin concentrations at the end of each state are stored in different objects. At the end of the *for* loop, for each cell, a time point is chosen at which the cyclins and DNA values are recomputed using the information stored in the objects earlier. These are the values which are output in the simulation plots in Figures 2 & 3. Cells in different states are output in separate files. More detailed documentation can be found in the source code itself.

**Source Code 2: Implementing the hybrid model used to generate the simulation curves in Figure 4 (Pages 29—71).** Additions to Source Code 1 were made to simulate a growing population of HUVEC cells from Day 0 to Day 10. Each cell is stored as an object containing information such as clock time at birth, clock time at division, mass at birth, etc. A simulated population of 500 cells is first generated at Day 0 (0 hours), and sorted by their clock time at division, in ascending order. The clock runs until the number of hours specified by the user. A *while* loop checks if the next cell’s clock time at division is less than the maximum clock time specified, and if so, generates two daughter cells whose clock time at birth is equal to the clock time at division of their parent cell. After these two daughter cells go through their own sequence of states, they are also stored as part of the population, according to their own clock time at division. After we find a parent cell whose clock time at division is more than the maximum clock time specified, the *while* loop stops executing. Now, because we have recorded the at-birth and at-division clock time for each cell, we can check to see how many cells are present on each successive day, which gives us the growth curve we are looking for. Furthermore, the code is also able to determine the cell cycle state of each cell selected on a particular day. This enables easy computation of the percentage of cells in G1, S & G2/M phases on each successive day. Again, the output is recorded in text files with the appropriate tabular format, which are then plotted using OriginPro8. More detailed documentation can be found in the source code itself.

// SOURCE CODE 1: HYBRID MODEL

#define _CRT_RAND_S

// Rajat Singhania, started on August 27, 2008; final documentation completed on September 22, 2010

// This program simulates the hybrid deterministic-stochastic mammalian cell cycle model.

// Using the Boolean table developed by JJT on Wednesday, Aug. 27, 2009 (as described in Table 1 of the paper)

// We have divided the cell cycle into 9 states, which have been defined as:

// State 1: G1a

// State 2: early G1b

// State 3: late G1b

// State 4: S

// State 5: G2

// State 6: PRO (Prophase)

// State 7: MET (Prometaphase/Metaphase)

// State 8: Anaphase

// State 9: Telophase

#include<iostream>

#include<fstream>

#include<sstream>

#include<vector>

#include<string>

#include<cmath>

#include<set>

#include<map>

using namespace std;

// User defined functions:

double calcCycA(double CycA, int state, double time); // calculating Cyclin A concentration using time spent in a State

double calcCycB(double CycB, int state, double time); // calculating Cyclin B concentration using time spent in a State

double calcCycE(double CycE, int state, double time); // calculating Cyclin E concentration using time spent in a State

double calcCycD(double CycD, double time); // calculating Cyclin D concentration using time spent in a given State

// calculating time needed to cross the Cyclin E level threshold needed to transition from State 2 (early G1b) to State 3 (late G1b)

double calcEarlyG1Time(double kde, double kse, double increment, double CycE, double mass, double t, double threshold, ofstream &);

// function to calculate the (deterministic) time needed to cross a threshold, as used in the transition from State 5 to 6, below

double calcDetTime(double init_Cyc, double kd, double ks);

// Gaussian random number generator

float box_muller(float m, float s); /* mean m, standard deviation s */

// Uniform random number generator

double randNumber();

// Kinetic Parameters...

double const k_sa_p = 5; // background synthesis rate

double const k_sa_pp = 6; // TFA synthesis rate

double const k_sa_ppp = 20; // TFB synthesis rate

double const k_da_p = 0.2; // background degradation rate

double const k_da_pp = 1.2; // Cdc20A degradation rate

double const k_da_ppp = 1.2; // Cdh1 degradation rate

double const k_sb_p = 2.5; // background synthesis rate

double const k_sb_pp = 6; // TFB synthesis rate

double const k_db_p = 0.2; // background degradation rate

double const k_db_pp = 1.2; // Cdc20B degradation rate

double const k_db_ppp = 0.3; // Cdh1 degradation rate

double const k_se_p = 0.02; // background synthesis rate

double const k_se_pp = 2; // TFE synthesis rate

double const k_de_p = 0.02; // background degradation rate

double const k_de_pp = 0.5; // SCF degradation rate

void main()

{

ofstream out("newHybridResults.txt"); // printing out (for cycles 501 to 32,500) the to-be-plotted output values for the simulation

ofstream out2("log_CycA.txt"); // each line records, for each cell, the concentration of Cyclin A at the beginning of each state

ofstream out3("cumulTimes.txt"); // each line records, for each cell, the cumulative time spent in the cycle by the end of each state

ofstream out4("cellCycles.txt"); // printing out (for cycles 501 to 32,500) the initial mass, division mass, and cycle time

ofstream out5("masses.txt"); // each line records, for each cell, the mass at the beginning/end of each state

ofstream out6("log_CycB.txt"); // each line records, for each cell, the concentration of Cyclin B at the beginning of each state

ofstream out7("log_CycE.txt"); // each line records, for each cell, the concentration of Cyclin E at the beginning of each state

ofstream out9("log_CycD.txt"); // each line records, for each cell, the concentration of Cyclin D at the beginning of each state

ofstream out10("cellAges.txt"); // each line records, for each cell, the time at which it was picked to record its simulation output values,

// the corresponding cell cycle phase (between 0 and 1), and the total cycle time

ofstream out11("delta_t.txt"); // see calcEarlyG1Time function implementation towards the bottom of the code

ofstream state1("G0.txt"); // recording the simulated G0 (G1a) cells that are also output in the file newHybridResults.txt

ofstream state2("eG1.txt"); // recording the simulated early G1b cells that are also output in the file newHybridResults.txt

ofstream state3("lG1.txt"); // recording the simulated late G1b cells that are also output in the file newHybridResults.txt

ofstream state4("S.txt"); // recording the simulated S cells that are also output in the file newHybridResults.txt

ofstream state5("G2.txt"); // recording the simulated G2 cells that are also output in the file newHybridResults.txt

ofstream state6("PRO.txt"); // recording the simulated Prophase cells that are also output in the file newHybridResults.txt

ofstream state7("MET.txt"); // recording the simulated Pro/Metaphase cells that are also output in the file newHybridResults.txt

ofstream state8("ANA.txt"); // recording the simulated Anaphase cells that are also output in the file newHybridResults.txt

ofstream state9("TEL.txt"); // recording the simulated Telophase cells that are also output in the file newHybridResults.txt

// printing the header line for each output file

out << "State\tDNA\tCycA\tCycE\tCycB\tCycD" << endl;

out4 << "Initial Mass\tDivision Mass\tCycle Time" << endl;

state1 << "State\tDNA\tCycA\tCycE\tCycB\tCycD" << endl;

state2 << "State\tDNA\tCycA\tCycE\tCycB\tCycD" << endl;

state3 << "State\tDNA\tCycA\tCycE\tCycB\tCycD" << endl;

state4 << "State\tDNA\tCycA\tCycE\tCycB\tCycD" << endl;

state5 << "State\tDNA\tCycA\tCycE\tCycB\tCycD" << endl;

state6 << "State\tDNA\tCycA\tCycE\tCycB\tCycD" << endl;

state7 << "State\tDNA\tCycA\tCycE\tCycB\tCycD" << endl;

state8 << "State\tDNA\tCycA\tCycE\tCycB\tCycD" << endl;

state9 << "State\tDNA\tCycA\tCycE\tCycB\tCycD" << endl;

double mass, CycA, CycB, CycD, CycE; // mass & Cyclin concentrations

double init_CycA, init_CycB, init_CycE, init_CycD; // initial Cyclin concentrations

double DNA, synthStartTime, synthDuration, synthStopTime; // DNA synthesis start time, duration, and end time

double initTime; // the initial time of the state picked for a simulated cell for final results/plotting

double initial_mass = 3; // initial mass used to start off the simulation

const double increment = 0.029; // the exponential rate at which the mass grows; unit: 1/hr.

// experimental/instrumental noise (modeled using a Gaussian distribution with the stated mean and standard deviations):

float mean = 1;

float deviation_cyclin_a = 0.15;

float deviation_cyclin_b = 0.15;

float deviation_cyclin_e = 0.15;

float deviation_cyclin_d = 0.2;

float deviation_dna = 0.03;

float deviation_mass = 0.033;

vector<double> stateTimes; // cumulative time spent at the end of each state, for each cell

// Helps in finding the state of each cell in Stage 2 of the simulation

vector<double> init_CycA_vec; // cyclin A concentration at the beginning of each state a single cell

vector<double> init_CycB_vec; // cyclin B concentration at the beginning of each state for each cell

vector<double> init_CycE_vec; // cyclin E concentration at the beginning of each state for each cell

vector<double> init_CycD_vec; // cyclin D concentration at the beginning of each state for each cell

// the variables for all the times recorded in the simulation; unit: hr.

// det: deterministic time; ran: random time

double time_S1, time_S2, time_S3, time_S4, time_S5, time_S6, time_S7, time_S8, time_S9, cumulTime;

double time_S1_det, time_S1_ran, time_S2_det, time_S2_ran, time_S3_det, time_S3_ran, time_S4_det, time_S4_ran;

double time_S5_det, time_S5_ran, time_S9_det, time_S9_ran;

double timeChosen, phase; // time & phase at which a cell is chosen to be plotted

double cellCycleTime;

double kse_two = k_se_p + k_se_pp; // overall synthesis rate for Cyclin E in State 2

// k_se_pp * B_TFE = k_se_pp in this state since B_TFE = 1

// Likewise in all the cases below...

double kde_two = k_de_p; // overall degradation rate for Cyclin E in State 2

double ksa_three = k_sa_p + k_sa_pp;// overall synthesis rate for Cyclin A in State 3

double kda_three = k_da_p; // overall degradation rate for Cyclin A in State 3

double ksb_five = k_sb_p + k_sb_pp; // overall synthesis rate for Cyclin B in State 5

double kdb_five = k_db_p; // overall degradation rate for Cyclin B in State 5

double ksb_eight = k_sb_p + k_sb_pp;// overall synthesis rate for Cyclin B in State 8

double kdb_eight = k_db_p + k_db_pp;// overall degradation rate for Cyclin B in State 8

double ksb_nine = k_sb_p; // overall synthesis rate for Cyclin B in State 9

double kdb_nine = k_db_p + k_db_pp + k_db_ppp;// overall degradation rate for Cyclin B in State 9

double init_t = 0; // time at the beginning of the delta t calculation for State 2 to 3 transition.

double steadystate_end_five_CycB = ksb_five/kdb_five; // the (fixed) steady state of CycB concentration, end of State 5

double steadystate_end_eight_CycB = ksb_eight/kdb_eight; // the (fixed) steady state of CycB concentration, end of State 8

double threshold_two_three = 80; // the fixed threshold for size control used in going from State 2 to 3

double threshold_three_four_CycA = 12.5; // the fixed threshold in CycA concentration needed to go over

// to cross from State 3 to State 4

double threshold_five_six_CycB = 0.5*steadystate_end_five_CycB; // the (fixed) threshold in CycB concentration needed to go over

// to cross from State 5 to State 6

double threshold_nine_end_CycB = 0.5*steadystate_end_eight_CycB;// the (fixed) threshold in CycB concentration needed to fall below

// to cross from State 9 to State 1

// defining the lambda's to be used in time_S#_ran calculations!!!

double lambda_one = 2;

double lambda_two = 0;

double lambda_three = 0.01;

double lambda_four = 1;

double lambda_five = 0.5;

double lambda_six = 0.75;

double lambda_seven = 1.5;

double lambda_eight = 0.5;

double lambda_nine = 0.025;

// initial cyclin concentrations...

init_CycA = 1;

init_CycB = 1;

init_CycE = 1;

init_CycD = 1;

// cyclin amounts (levels), i.e., concentrations times mass

double Amt_CycB, Amt_CycA, Amt_CycE, Amt_CycD;

mass = initial_mass; // mass at beginning of state 1

for (int j = 1; j <= 32500; j++) // iterating through the cell cycle 32,500 times

{

if (j > 500) out4 << mass << "\t"; // discarding the first 500 cells to get rid of the initial conditions' effects

out5 << mass << "\t";

// clearing out the data vectors

init_CycA_vec.clear();

init_CycB_vec.clear();

init_CycE_vec.clear();

init_CycD_vec.clear();

stateTimes.clear();

CycA = init_CycA;

CycB = init_CycB;

CycE = init_CycE;

CycD = init_CycD;

init_CycA_vec.push_back(CycA); // coming from end of state 9 calculations!

init_CycB_vec.push_back(CycB);

init_CycE_vec.push_back(CycE);

init_CycD_vec.push_back(CycD);

out2 << CycA << "\t";

out6 << CycB << "\t";

out7 << CycE << "\t";

out9 << CycD << "\t";

cumulTime = 0; // at the beginning of a new cell iteration

out3 << cumulTime << "\t";

time_S1_det = 0;

time_S1_ran = lambda_one*log(1/(1-randNumber()));

time_S1 = time_S1_det + time_S1_ran;

mass = mass*exp(increment * time_S1);

out5 << mass << "\t";

cumulTime += time_S1;

stateTimes.push_back(cumulTime); // time spent in state 1...

out3 << cumulTime << "\t";

CycA = calcCycA(CycA, 1, time_S1); // calculating Cyclin concentrations at the end of the state!!!

CycB = calcCycB(CycB, 1, time_S1); // calculating Cyclin concentrations at the end of the state!!!

CycE = calcCycE(CycE, 1, time_S1); // calculating Cyclin concentrations at the end of the state!!!

CycD = calcCycD(CycD, time_S1);

out2 << CycA << "\t";

out6 << CycB << "\t";

out7 << CycE << "\t";

out9 << CycD << "\t";

init_CycA_vec.push_back(CycA);

init_CycB_vec.push_back(CycB);

init_CycE_vec.push_back(CycE);

init_CycD_vec.push_back(CycD);

if (CycE * mass >= threshold_two_three) // first checking if the Cyclin E level has crossed the specified threshold

time_S2 = 0;

else

time_S2 = calcEarlyG1Time(kde_two, kse_two, increment, CycE, mass, init_t, threshold_two_three, out11); // early G1

// calculating time needed to cross the Cyclin E level threshold needed to transition from State 2 (early G1b) to

// State 3 (late G1b)

cumulTime += time_S2;

mass = mass*exp(increment*time_S2); // mass at end of state 2, beginning of state 3

out5 << mass << "\t";

stateTimes.push_back(cumulTime);

out3 << cumulTime << "\t";

CycA = calcCycA(CycA, 2, time_S2); // calculating Cyclin concentrations at the end of the state!!!

CycB = calcCycB(CycB, 2, time_S2); // calculating Cyclin concentrations at the end of the state!!!

CycE = calcCycE(CycE, 2, time_S2); // calculating Cyclin concentrations at the end of the state!!!

CycD = calcCycD(CycD, time_S2);

out11 << "Mass times the concentration of Cyclin E is: " << mass*CycE << endl;

out2 << CycA << "\t";

out6 << CycB << "\t";

out7 << CycE << "\t";

out9 << CycD << "\t";

init_CycA_vec.push_back(CycA);

init_CycB_vec.push_back(CycB);

init_CycE_vec.push_back(CycE);

init_CycD_vec.push_back(CycD);

if (CycA >= threshold_three_four_CycA) // first checking if the Cyclin A concentration has crossed the specified threshold

{

time_S3_det = 0;

}

else

{

//time_S3_det = calcDetTime(CycA, kda_three, ksa_three); // was valid when trying to reach half-of-steady-state

time_S3_det = (double(1)/kda_three)*log((CycA - (ksa_three/kda_three))/((threshold_three_four_CycA - (ksa_three/kda_three))));

// currently trying to reach a specific, constant threshold, threshold_three_four_CycA = 12.5

}

time_S3_ran = lambda_three*log(1/(1-randNumber()));

time_S3 = time_S3_det + time_S3_ran;

mass = mass*exp(increment*time_S3); // mass at end of state 3, beginning of state 4

out5 << mass << "\t";

cumulTime += time_S3;

stateTimes.push_back(cumulTime);

out3 << cumulTime << "\t";

CycA = calcCycA(CycA, 3, time_S3); // calculating Cyclin concentrations at the end of the state!!!

CycB = calcCycB(CycB, 3, time_S3); // calculating Cyclin concentrations at the end of the state!!!

CycE = calcCycE(CycE, 3, time_S3); // calculating Cyclin concentrations at the end of the state!!!

CycD = calcCycD(CycD, time_S3);

out2 << CycA << "\t";

out6 << CycB << "\t";

out7 << CycE << "\t";

out9 << CycD << "\t";

init_CycA_vec.push_back(CycA);

init_CycB_vec.push_back(CycB);

init_CycE_vec.push_back(CycE);

init_CycD_vec.push_back(CycD);

// Starting State 4 calculations

synthStartTime = cumulTime;

time_S4_det = 7;

time_S4_ran = lambda_four*log(1/(1-randNumber()));

time_S4 = time_S4_det + time_S4_ran;

synthDuration = time_S4;

cumulTime += time_S4;

mass = mass*exp(increment*time_S4); // mass at end of state 4, beginning of state 5

out5 << mass << "\t";

stateTimes.push_back(cumulTime);

out3 << cumulTime << "\t";

CycA = calcCycA(CycA, 4, time_S4); // calculating Cyclin concentrations at the end of the state!!!

CycB = calcCycB(CycB, 4, time_S4); // calculating Cyclin concentrations at the end of the state!!!

CycE = calcCycE(CycE, 4, time_S4); // calculating Cyclin concentrations at the end of the state!!!

CycD = calcCycD(CycD, time_S4);

out2 << CycA << "\t";

out6 << CycB << "\t";

out7 << CycE << "\t";

out9 << CycD << "\t";

init_CycA_vec.push_back(CycA);

init_CycB_vec.push_back(CycB);

init_CycE_vec.push_back(CycE);

init_CycD_vec.push_back(CycD);

synthStopTime = cumulTime;

// State 5 calculations

if (CycB >= threshold_five_six_CycB) // first checking if the Cyclin B concentration has crossed the specified threshold

{

time_S5_det = 0;

}

else

{

time_S5_det = calcDetTime(CycB, kdb_five, ksb_five); // Used function valid as we are trying to go over

// half-of-steady-state of Cyclin B at the end of State 5

}

time_S5_ran = lambda_five*log(1/(1-randNumber()));

time_S5 = time_S5_det + time_S5_ran;

cumulTime += time_S5;

mass = mass*exp(increment*time_S5); // mass at end of state 5, beginning of state 6

out5 << mass << "\t";

stateTimes.push_back(cumulTime);

out3 << cumulTime << "\t";

CycA = calcCycA(CycA, 5, time_S5); // calculating Cyclin concentrations at the end of the state!!!

CycB = calcCycB(CycB, 5, time_S5); // calculating Cyclin concentrations at the end of the state!!!

CycE = calcCycE(CycE, 5, time_S5); // calculating Cyclin concentrations at the end of the state!!!

CycD = calcCycD(CycD, time_S5);

out2 << CycA << "\t";

out6 << CycB << "\t";

out7 << CycE << "\t";

out9 << CycD << "\t";

init_CycA_vec.push_back(CycA);

init_CycB_vec.push_back(CycB);

init_CycE_vec.push_back(CycE);

init_CycD_vec.push_back(CycD);

time_S6 = lambda_six*log(1/(1-randNumber())); // Prophase

cumulTime += time_S6;

mass = mass*exp(increment*time_S6); // mass at end of state 6, beginning of state 7

out5 << mass << "\t";

stateTimes.push_back(cumulTime);

out3 << cumulTime << "\t";

CycA = calcCycA(CycA, 6, time_S6); // calculating Cyclin concentrations at the end of the state!!!

CycB = calcCycB(CycB, 6, time_S6); // calculating Cyclin concentrations at the end of the state!!!

CycE = calcCycE(CycE, 6, time_S6); // calculating Cyclin concentrations at the end of the state!!!

CycD = calcCycD(CycD, time_S6);

out2 << CycA << "\t";

out6 << CycB << "\t";

out7 << CycE << "\t";

out9 << CycD << "\t";

init_CycA_vec.push_back(CycA);

init_CycB_vec.push_back(CycB);

init_CycE_vec.push_back(CycE);

init_CycD_vec.push_back(CycD);

time_S7 = lambda_seven*log(1/(1-randNumber())); // Prometaphase/Metaphase

cumulTime += time_S7;

mass = mass*exp(increment*time_S7); // mass at end of state 7, beginning of state 8

out5 << mass << "\t";

stateTimes.push_back(cumulTime);

out3 << cumulTime << "\t";

CycA = calcCycA(CycA, 7, time_S7); // calculating Cyclin concentrations at the end of the state!!!

CycB = calcCycB(CycB, 7, time_S7); // calculating Cyclin concentrations at the end of the state!!!

CycE = calcCycE(CycE, 7, time_S7); // calculating Cyclin concentrations at the end of the state!!!

CycD = calcCycD(CycD, time_S7);

out2 << CycA << "\t";

out6 << CycB << "\t";

out7 << CycE << "\t";

out9 << CycD << "\t";

init_CycA_vec.push_back(CycA);

init_CycB_vec.push_back(CycB);

init_CycE_vec.push_back(CycE);

init_CycD_vec.push_back(CycD);

time_S8 = lambda_eight*log(1/(1-randNumber())); // Anaphase

cumulTime += time_S8;

mass = mass*exp(increment*time_S8); // mass at end of state 8, beginning of state 9

out5 << mass << "\t";

stateTimes.push_back(cumulTime);

out3 << cumulTime << "\t";

CycA = calcCycA(CycA, 8, time_S8); // calculating Cyclin concentrations at the end of the state!!!

CycB = calcCycB(CycB, 8, time_S8); // calculating Cyclin concentrations at the end of the state!!!

CycE = calcCycE(CycE, 8, time_S8); // calculating Cyclin concentrations at the end of the state!!!

CycD = calcCycD(CycD, time_S8);

out2 << CycA << "\n";

out6 << CycB << "\n";

out7 << CycE << "\n";

out9 << CycD << "\n";

init_CycA_vec.push_back(CycA);

init_CycB_vec.push_back(CycB);

init_CycE_vec.push_back(CycE);

init_CycD_vec.push_back(CycD);

if (CycB <= threshold_nine_end_CycB) // first checking if the Cyclin B concentration has fallen below the specified threshold

{

time_S9_det = 0;

}

else

{

time_S9_det = (double(1)/kdb_nine)*log((CycB - (ksb_nine/kdb_nine))/((0.5*(ksb_eight/kdb_eight) - (ksb_nine/kdb_nine))));

// Used function valid as we are trying to go under half-of-steady-state of Cyclin B at the end of State 8

}

time_S9_ran = lambda_nine*log(1/(1-randNumber()));

time_S9 = time_S9_det + time_S9_ran;

cumulTime += time_S9;

mass = mass*exp(increment*time_S9); // mass at end of state 9

out5 << mass << "\t";

stateTimes.push_back(cumulTime);

out3 << cumulTime << "\n";

CycA = calcCycA(CycA, 9, time_S9); // calculating Cyclin concentrations at the end of the state!!!

CycB = calcCycB(CycB, 9, time_S9); // calculating Cyclin concentrations at the end of the state!!!

CycE = calcCycE(CycE, 9, time_S9); // calculating Cyclin concentrations at the end of the state!!!

CycD = calcCycD(CycD, time_S9);

// setting the initial Cyclin concentrations for the next cycle using the concentrations at the end of the current cycle

init_CycA = CycA;

init_CycB = CycB;

init_CycE = CycE;

init_CycD = CycD;

if (j > 500) out4 << mass << "\t"; // discard first 500 cells to get rid of initial conditions

mass = mass*0.5*box_muller(mean, deviation_mass); // divide the mass by half (and add Gaussian noise) at end of the current cycle

out5 << mass << "\n";

cellCycleTime = cumulTime; // the cumulative time at the end of State 9 is the cell cycle time

if (j > 500) out4 << cellCycleTime << endl; // again, discard first 500 cells to get rid of initial conditions

phase = log(double(2/(2-randNumber())))/log(double(2)); // number between 0 and 1, chosen as described in the paper

timeChosen = phase*cellCycleTime; // time at which a cell is chosen to be plotted

out10 << timeChosen << "\t" << phase << "\t" << cellCycleTime << "\t";

int currState; // current state, used right below...

int flag; // used right below...

// iterating through all the 9 states for a given cell by using the information stored in the vectors above

for (int i = 0; i < stateTimes.size(); i++)

{

flag = 0;

// check if the time chosen for the cell is less than the time at the end of a state, as has already been recorded earlier...

if (timeChosen <= stateTimes[i])

{

currState = i+1; // ...in that case, specify the current state as the output state

flag = 1; // indicator that the above condition has been met

// finding the starting time of the current state which is going to be simulated/output

if (currState == 1) { initTime = 0; }

else if (currState > 1) { initTime = stateTimes[i-1]; }

// calculating Cyclin amounts according to the current state, time spent in the state, and mass at the chosen time

Amt_CycA = calcCycA(init_CycA_vec[i], currState, timeChosen-initTime)*(initial_mass*exp(increment*timeChosen));

Amt_CycB = calcCycB(init_CycB_vec[i], currState, timeChosen-initTime)*(initial_mass*exp(increment*timeChosen));

Amt_CycE = calcCycE(init_CycE_vec[i], currState, timeChosen-initTime)*(initial_mass*exp(increment*timeChosen));

Amt_CycD = calcCycD(init_CycD_vec[i], timeChosen-initTime)*(initial_mass*exp(increment*timeChosen));

out10 << j << endl;

// adding Gaussian noise to the Cyclin amounts calculated above

Amt_CycA *= box_muller(mean, deviation_cyclin_a);

Amt_CycB *= box_muller(mean, deviation_cyclin_b);

Amt_CycE *= box_muller(mean, deviation_cyclin_e);

Amt_CycD *= box_muller(mean, deviation_cyclin_d);

// measuring DNA content:

if (timeChosen <= synthStartTime)

{

DNA = 1;

}

else if (timeChosen >= synthStopTime)

{

DNA = 2;

}

else

{

DNA = 1 + (double(double(1)/synthDuration))*(timeChosen - synthStartTime);

// function to calculate DNA content if the cell is in State 4

}

DNA *= box_muller(mean, deviation_dna); // adding Gaussian noise to DNA content

if (j > 500) // discarding the first 500 cells to get rid of the effects of the initial conditions

{

out << currState << "\t" << DNA << "\t" << Amt_CycA << "\t" << Amt_CycE << "\t" << Amt_CycB << "\t" << Amt_CycD << endl;

// outputting the results for each state in separate files, as well

if (currState == 1)

state1 << currState << "\t" << DNA << "\t" << Amt_CycA << "\t" << Amt_CycE << "\t" << Amt_CycB << "\t" << Amt_CycD << endl;

else if (currState == 2)

state2 << currState << "\t" << DNA << "\t" << Amt_CycA << "\t" << Amt_CycE << "\t" << Amt_CycB << "\t" << Amt_CycD << endl;

else if (currState == 3)

state3 << currState << "\t" << DNA << "\t" << Amt_CycA << "\t" << Amt_CycE << "\t" << Amt_CycB << "\t" << Amt_CycD << endl;

else if (currState == 4)

state4 << currState << "\t" << DNA << "\t" << Amt_CycA << "\t" << Amt_CycE << "\t" << Amt_CycB << "\t" << Amt_CycD << endl;

else if (currState == 5)

state5 << currState << "\t" << DNA << "\t" << Amt_CycA << "\t" << Amt_CycE << "\t" << Amt_CycB << "\t" << Amt_CycD << endl;

else if (currState == 6)

state6 << currState << "\t" << DNA << "\t" << Amt_CycA << "\t" << Amt_CycE << "\t" << Amt_CycB << "\t" << Amt_CycD << endl;

else if (currState == 7)

state7 << currState << "\t" << DNA << "\t" << Amt_CycA << "\t" << Amt_CycE << "\t" << Amt_CycB << "\t" << Amt_CycD << endl;

else if (currState == 8)

state8 << currState << "\t" << DNA << "\t" << Amt_CycA << "\t" << Amt_CycE << "\t" << Amt_CycB << "\t" << Amt_CycD << endl;

else if (currState == 9)

state9 << currState << "\t" << DNA << "\t" << Amt_CycA << "\t" << Amt_CycE << "\t" << Amt_CycB << "\t" << Amt_CycD << endl;

}

break; // once the state has been found at which to plot the cell, exit this loop

}

}

if (flag == 0) { cout << "WOOPS!!" << endl; } // if somehow the time chosen for a cycle to be simulated at

// is not less than the total cell cycle time

}

}

// calculating Cyclin A concentration using time spent in a State, at the End of the state

double calcCycA(double CycA, int state, double time) // CycA is the Cyclin A concentration at the Beginning of the input state

{

int TF_e, Cdc20_a, Cdh1, TF_b; // Cyclin A regulators whose values are stored below according to the Boolean state table

switch(state)

{

case 1:

// G0

TF_e = 0;

TF_b = 0;

Cdc20_a = 0;

Cdh1 = 1;

break;

case 2:

// early G1

TF_e = 1;

TF_b = 0;

Cdc20_a = 0;

Cdh1 = 1;

break;

case 3:

// late G1

TF_e = 1;

TF_b = 0;

Cdc20_a = 0;

Cdh1 = 0;

break;

case 4:

// S

TF_e = 1;

TF_b = 0;

Cdc20_a = 0;

Cdh1 = 0;

break;

case 5:

// G2

TF_e = 1;

TF_b = 1;

Cdc20_a = 0;

Cdh1 = 0;

break;

case 6:

// Prophase

TF_e = 0;

TF_b = 1;

Cdc20_a = 0;

Cdh1 = 0;

break;

case 7:

// Prometaphase/Metaphase

TF_e = 0;

TF_b = 1;

Cdc20_a = 1;

Cdh1 = 0;

break;

case 8:

// Anaphase

TF_e = 0;

TF_b = 1;

Cdc20_a = 1;

Cdh1 = 0;

break;

case 9:

// Telophase

TF_e = 0;

TF_b = 0;

Cdc20_a = 1;

Cdh1 = 1;

break;

}

double k_sa = k_sa_p + k_sa_pp * TF_e + k_sa_ppp * TF_b; // overall Cyclin A synthesis rate

double k_da = k_da_p + k_da_pp * Cdc20_a + k_da_ppp * Cdh1; // overall Cyclin A degradation rate

CycA = (k_sa/k_da) + (CycA - (k_sa/k_da))*exp(-1*k_da*time); // CycA concentration, solved using an explicit equation, without need to integrate

return CycA;

}

// calculating Cyclin D concentration using time spent in a State

double calcCycD(double CycD, double time)

{

double k_sd = 0.2;

double k_dd = 0.2;

CycD = (k_sd/k_dd) + (CycD - (k_sd/k_dd))*exp(-1*k_dd*time);

return CycD;

}

// calculating Cyclin B concentration using time spent in a State, at the End of the state

double calcCycB(double CycB, int state, double time) // CycB is the Cyclin B concentration at the Beginning of the input state

{

int TF_b, Cdc20_b, Cdh1; // Cyclin B regulators whose values are stored below according to the Boolean state table

switch(state)

{

case 1:

// G0

TF_b = 0;

Cdc20_b = 0;

Cdh1 = 1;

break;

case 2:

// early G1

TF_b = 0;

Cdc20_b = 0;

Cdh1 = 1;

break;

case 3:

// late G1

TF_b = 0;

Cdc20_b = 0;

Cdh1 = 0;

break;

case 4:

// S

TF_b = 0;

Cdc20_b = 0;

Cdh1 = 0;

break;

case 5:

// G2

TF_b = 1;

Cdc20_b = 0;

Cdh1 = 0;

break;

case 6:

// Prophase

TF_b = 1;

Cdc20_b = 0;

Cdh1 = 0;

break;

case 7:

// Prometaphase/Metaphase

TF_b = 1;

Cdc20_b = 0;

Cdh1 = 0;

break;

case 8:

// Anaphase

TF_b = 1;

Cdc20_b = 1;

Cdh1 = 0;

break;

case 9:

// Telophase

TF_b = 0;

Cdc20_b = 1;

Cdh1 = 1;

break;

}

double k_sb = k_sb_p + k_sb_pp * TF_b; // overall Cyclin B synthesis rate

double k_db = k_db_p + k_db_pp * Cdc20_b + k_db_ppp * Cdh1; // overall Cyclin B degradation rate

CycB = (k_sb/k_db) + (CycB - k_sb/k_db)*exp(-1*k_db*time); // CycB concentration, solved using an explicit equation, without need to integrate

return CycB;

}

// calculating Cyclin E concentration using time spent in a State, at the End of the state

double calcCycE(double CycE, int state, double time) // CycE is the Cyclin E concentration at the Beginning of the input state

{

int TF_e, SCF; // Cyclin E regulators whose values are stored below according to the Boolean state table

switch(state)

{

case 1:

// G0

TF_e = 0;

SCF = 0;

break;

case 2:

// early G1

TF_e = 1;

SCF = 0;

break;

case 3:

// late G1

TF_e = 1;

SCF = 0;

break;

case 4:

// S

TF_e = 1;

SCF = 1;

break;

case 5:

// G2

TF_e = 1;

SCF = 1;

break;

case 6:

// Prophase

TF_e = 0;

SCF = 1;

break;

case 7:

// Prometaphase/Metaphase

TF_e = 0;

SCF = 1;

break;

case 8:

// Anaphase

TF_e = 0;

SCF = 1;

break;

case 9:

// Telophase

TF_e = 0;

SCF = 1;

break;

}

double k_se = k_se_p + k_se_pp * TF_e; // overall Cyclin E synthesis rate

double k_de = k_de_p + k_de_pp * SCF; // overall Cyclin E degradation rate

CycE = (k_se/k_de) + (CycE - k_se/k_de)*exp(-1*k_de*time); // CycE concentration, solved using an explicit equation, without need to integrate

return CycE;

}

// calculating time needed to cross the Cyclin E level threshold needed to transition from State 2 (early G1b) to State 3 (late G1b)

double calcEarlyG1Time(double kde, double kse, double increment, double CycE, double mass, double t, double threshold, ofstream &out11)

{

// w/Exponential growth

// Implementing the Newton-Rhapson method to find the time t @ which F(t) = (Cyclin E * mass) - threshold = 0

double CycE_ss = kse/kde;

double neg_F_t, FP_t;

double delta_t = 0;

double abs_delta_t;

bool done = false;

int counter = 0;

out11 << "=================================" << endl;

while (done == false)

{

counter++;

if (counter <= 100)

{

neg_F_t = (-1*CycE_ss) + (CycE_ss - CycE)*(exp(-1*kde*t)) + (threshold*exp(-1*increment*t)/mass);

FP_t = kde*(CycE_ss - CycE)*(exp(-1*kde*t)) + (threshold*increment*exp(-1*increment*t)/mass);

delta_t = neg_F_t/FP_t;

out11 << delta_t << endl;

if (delta_t < 0) { abs_delta_t = -1*delta_t; } else {abs_delta_t = delta_t; }

if (abs_delta_t <= pow(double(10), double(-6)))

{

done = true;

}

else

{

t = t + delta_t;

done = false;

}

}

else break;

}

if (done == true)

return t;

else cout << "The 'mass times concentration' iterations failed to converge!!" << endl;

}

// function to calculate the (deterministic) time needed to cross a threshold, as used in the transition from State 5 to 6, below

double calcDetTime(double init_Cyc, double kd, double ks)

{

double detTime; // deterministic time spent in a particular state

double one = 1, two = 2;

detTime = (one/kd)*log(two*(one - (kd*init_Cyc/ks)));

return detTime;

}

/* boxmuller.c Implements the Polar form of the Box-Muller

Transformation

(c) Copyright 1994, Everett F. Carter Jr.

Permission is granted by the author to use

this software for any application provided this

copyright notice is preserved.

ftp://ftp.taygeta.com/pub/c/boxmuller.c

// Note by Rajat Singhania: made some cosmetic changes of my own to produce the code below.

*/

// Gaussian random number generator

float box_muller(float m, float s) /* mean m, standard deviation s */

{

float x1, x2, w, y1, y2;

do {

x1 = 2.0 * randNumber() - 1.0;

x2 = 2.0 * randNumber() - 1.0;

w = x1 * x1 + x2 * x2;

} while ( w >= 1.0 );

w = sqrt( (-2.0 * log( w ) ) / w );

y1 = x1 * w;

y2 = x2 * w;

float toReturn1 = m + y1 * s;

float toReturn2 = m + y2 * s;

if (toReturn1 > 0)

return toReturn1;

else if (toReturn2 > 0)

return toReturn2;

}

// Uniform random number generator to get values between 0 and 1

double randNumber()

{

double value = rand()/((double)RAND_MAX + 1);

return value;

}

// SOURCE CODE 2: GROWTH CURVE

#define _CRT_RAND_S

// Rajat Singhania, started on Wednesday, August 27, 2008; final documentation completed on March 23, 2010

// further documentation may be obtained from the base model

// Edited on January 31, 2010 with updated parameters/settings from the project 'JWJ_Hybrid_Model'

// This program simulates a flow cyometry plots-based time profile data

// using the hybrid continuous-discrete & deterministic-stochastic mammalian cell cycle model.

// Using the Boolean table developed by JJT on Wednesday, Aug. 27 (as described in Table 1 of the paper)

// There are still nine cell cycle states, which have been defined as:

// State 1: G1a

// State 2: early G1b

// State 3: late G1b

// State 4: S

// State 5: G2

// State 6: PRO (Prophase)

// State 7: MET (Prometaphase/Metaphase)

// State 8: Anaphase

// State 9: Telophase

#include<iostream>

#include<fstream>

#include<sstream>

#include<vector>

#include<string>

#include<cmath>

#include<set>

#include<map>

using namespace std;

class Cell

{

public:

int cellNumber;

vector<double> stateTimes; // cumulative time spent at the end of each state, for each "cell".

// Helps in finding the state of each "cell".

// vectors for cyclin concentrations at the beginning of each state for the cell:

vector<double> initCycAvec;

vector<double> initCycBvec;

vector<double> initCycEvec;

double birthMass;

double divMass;

double clockTimeAtBgng; // will be 0 for first 5000 cells...

double clockTimeAtDiv; //.......... key for the multimap

double cycleTime;

};

// User defined functions:

double calcCycA(double CycA, int state, double time); // calculating Cyclin A concentration using time spent in a State

double calcCycB(double CycB, int state, double time); // calculating Cyclin B concentration using time spent in a State

double calcCycE(double CycE, int state, double time); // calculating Cyclin E concentration using time spent in a State

double calcCycD(double CycD, double time); // calculating Cyclin D concentration using time spent in a given State

// calculating time needed to cross the Cyclin E level threshold needed to transition from State 2 (early G1b) to State 3 (late G1b)

double calcEarlyG1Time(double kde, double kse, double increment, double CycE, double mass, double t, double threshold, ofstream &);

// function to calculate the (deterministic) time needed to cross a threshold, as used in the transition from State 5 to 6, below

double calcDetTime(double init_Cyc, double kd, double ks);

// Gaussian random number generator

float box_muller(float m, float s); /* mean m, standard deviation s */

// Uniform random number generator

double randNumber();

// function to output flow cytometry plots for each cyclin on all the desired days

void outputFlowCytometry(double timeChosen, double initial_mass, vector<double>& state_Times, vector<double>& init_CycA_vec, vector<double>& init_CycB_vec, vector<double> init_CycE_vec, ofstream &day );

// Parameters....

double const k_sa_p = 5; // background synthesis rate

double const k_sa_pp = 6; // TFA synthesis rate

double const k_sa_ppp = 20; // TFB synthesis rate

double const k_da_p = 0.2; // background degradation rate

double const k_da_pp = 1.2; // Cdc20A degradation rate

double const k_da_ppp = 1.2; // Cdh1 degradation rate

double const k_sb_p = 2.5; // background synthesis rate

double const k_sb_pp = 6; // TFB synthesis rate

double const k_db_p = 0.2; // background degradation rate

double const k_db_pp = 1.2; // Cdc20B degradation rate

double const k_db_ppp = 0.3; // Cdh1 degradation rate

double const k_se_p = 0.02; // background synthesis rate

double const k_se_pp = 2; // TFE synthesis rate

double const k_de_p = 0.02; // background degradation rate

double const k_de_pp = 0.5; // SCF degradation rate

multimap<double, Cell> cellPopulation;

multimap<double, Cell>::iterator cellIter;

multimap<double, double>::iterator iter;

void main()

{

ofstream out("growthHybridResults_Day0_paper.txt");

ofstream out2("log_CycA_paper.txt");

ofstream out3("cumulTimes_paper.txt");

ofstream out4("cellCycles_paper.txt");

ofstream out5("masses_paper.txt");

ofstream out6("log_CycB_paper.txt");

ofstream out7("log_CycE_paper.txt");

// ofstream out9("log_CycD_paper.txt");

ofstream out10("cellAges_paper.txt");

ofstream out11("delta_t_paper.txt");

ofstream out13("cellPopulation_paper.txt");

ofstream out14("lineages_paper.txt");

ofstream out15("daily_population_count_paper.txt");

ofstream out16("G0_lambdas_paper.txt");

ofstream out17("population_size_paper.txt");

ofstream out18("iterations_paper.txt");

ofstream day1("growthHybridResults_Day1_paper.txt");

ofstream day2("growthHybridResults_Day2_paper.txt");

ofstream day3("growthHybridResults_Day3_paper.txt");

ofstream day4("growthHybridResults_Day4_paper.txt");

ofstream day5("growthHybridResults_Day5_paper.txt");

ofstream day6("growthHybridResults_Day6_paper.txt");

ofstream day7("growthHybridResults_Day7_paper.txt");

ofstream day8("growthHybridResults_Day8_paper.txt");

ofstream day9("growthHybridResults_Day9_paper.txt");

ofstream day10("growthHybridResults_Day10_paper.txt");

int choice;

double Hill_Coeff;

double N_nought, N_one;

int Init_Popn;

double numerator;

double Hours;

cout << "Enter 1 for a Hill function simulation, or 2 for a Hyperbolic tan function simulation: ";

cin >> choice; // entered 2 for paper simulation

cout << "Enter the number of hours for the simulation: ";

cin >> Hours; // entered 240 for paper simulation

cout << "\nEnter the numerator for the growth simulation: ";

cin >> numerator; // entered 0.5 for paper simulation; this is p_0 in Eq. [3] in the paper

cout << "\nEnter N_nought for the growth simulation: ";

cin >> N_nought; // entered 11,000 for paper simulation

if (choice == 1)

{

cout << "\nEnter the Hill Coefficient for the growth simulation: ";

cin >> Hill_Coeff;

}

else

{

cout << "\nEnter N_one for the growth simulation: ";

cin >> N_one; // entered 500 for paper simulation

}

cout << "\nEnter the initial population: ";

cin >> Init_Popn; // entered 500 for paper simulation

cout << endl;

out << "State\tDNA\tCycA\tCycE\tCycB" << endl;

day1 << "State\tDNA\tCycA\tCycE\tCycB" << endl;

day2 << "State\tDNA\tCycA\tCycE\tCycB" << endl;

day3 << "State\tDNA\tCycA\tCycE\tCycB" << endl;

day4 << "State\tDNA\tCycA\tCycE\tCycB" << endl;

day5 << "State\tDNA\tCycA\tCycE\tCycB" << endl;

day6 << "State\tDNA\tCycA\tCycE\tCycB" << endl;

day7 << "State\tDNA\tCycA\tCycE\tCycB" << endl;

day8 << "State\tDNA\tCycA\tCycE\tCycB" << endl;

day9 << "State\tDNA\tCycA\tCycE\tCycB" << endl;

day10 << "State\tDNA\tCycA\tCycE\tCycB" << endl;

double mass, CycA, CycB, CycE;

double init_CycA, init_CycB, init_CycE;

int currState = 0, flag; // there are 9 states in our model

double DNA = 1, synthStartTime, synthDuration, synthStopTime;

double initTime;

double initial_mass = 3;

const double increment = 0.029;

// experimental/instrumental noise...

float mean = 1;

float deviation_cyclin_a = 0.15;

float deviation_cyclin_b = 0.15;

float deviation_cyclin_e = 0.15;

float deviation_cyclin_d = 0.2;

float deviation_dna = 0.03;

float deviation_mass = 0.033;

vector<double> state_Times; // cumulative time spent at the end of each state, for each "cell".

// Helps in finding the state of each "cell" in Stage 2 of the simulation.

vector<double> init_CycA_vec;

vector<double> init_CycB_vec;

vector<double> init_CycE_vec;

double time_S1, time_S2, time_S3, time_S4, time_S5, time_S6, time_S7, time_S8, time_S9, cumulTime;

double time_S1_det, time_S1_ran, time_S2_det, time_S2_ran, time_S3_det, time_S3_ran, time_S4_det, time_S4_ran;

double time_S5_det, time_S5_ran, time_S9_det, time_S9_ran;

double timeChosen, phase, cellCycleTime;

double kse_two = k_se_p + k_se_pp;

double kde_two = k_de_p;

double ksa_two = k_sa_p + k_sa_pp;

double kda_two = k_da_p + k_da_ppp;

double ksa_three = k_sa_p + k_sa_pp;

double kda_three = k_da_p;

double ksb_four = k_sb_p;

double kdb_four = k_db_p;

double ksb_five = k_sb_p + k_sb_pp;

double kdb_five = k_db_p;

double ksb_eight = k_sb_p + k_sb_pp;

double kdb_eight = k_db_p + k_db_pp;

double ksb_nine = k_sb_p;

double kdb_nine = k_db_p + k_db_pp + k_db_ppp;

double init_t = 0; // time at the beginning of the delta t calculation for State 2 to 3 transition.

double threshold_two_three = 80; // size control

double steadystate_end_two_CycA = ksa_two/kda_two;

double steadystate_end_three_CycA = ksa_three/kda_three;

double steadystate_end_four_CycB = ksb_four/kdb_four;

double steadystate_end_five_CycB = ksb_five/kdb_five;

double steadystate_eight_CycB = ksb_eight/kdb_eight;

double threshold_three_four_CycA = 12.5; // 0.5*steadystate_end_three_CycA;

double threshold_five_six_CycB = 0.5*steadystate_end_five_CycB;

double threshold_nine_end = 0.5*steadystate_eight_CycB;

// defining the lambda's to be used in time_S#_ran calculations!!!

double lambda_one = 2;

double lambda_two = 0;

double lambda_three = 0.01;

double lambda_four = 1;

double lambda_five = 0.5;

double lambda_six = 0.75;

double lambda_seven = 1.5;

double lambda_eight = 0.5;

double lambda_nine = 0.025;

init_CycA = 1;

init_CycB = 1;

init_CycE = 1;

// init_CycD = 1;

out10 << "Time Chosen\tPhase\tCycle Time" << endl;

double Amt_CycB = 0;

double Amt_CycA = 0;

double Amt_CycE = 0;

// double Amt_CycD;

int cellNum = 0;

double clock_time_at_div, clock_time_at_birth;

double next_clock_time_at_div;

double next_clock_time_at_birth;

mass = initial_mass; // mass at beginning of state 1

double birth_mass;

CycA = init_CycA;

CycB = init_CycB;

CycE = init_CycE;

// CycD = init_CycD;

double mass_500;

double CycA_500;

double CycB_500;

double CycE_500;

for (int j = 1; j <= 500 + Init_Popn; j++)

{

if (j == 501) { mass_500 = mass; CycA_500 = CycA; CycB_500 = CycB; CycE_500 = CycE; } // since the first 500 cells are discarded

// to get rid of initial condition effects

if (j > 500)

{

cellNum++;

mass = mass_500;

CycA = CycA_500;

CycB = CycB_500;

CycE = CycE_500;

}

birth_mass = mass;

// out4 << birth_mass << "\t";

init_CycA_vec.clear();

init_CycB_vec.clear();

init_CycE_vec.clear();

// init_CycD_vec.clear();

state_Times.clear();

init_CycA_vec.push_back(CycA); // coming from end of state 9 calculations!

init_CycB_vec.push_back(CycB);

init_CycE_vec.push_back(CycE);

// init_CycD_vec.push_back(CycD);

out5 << mass << "\t";

out2 << CycA << "\t";

out6 << CycB << "\t";

out7 << CycE << "\t";

// out9 << CycD << "\t";

cumulTime = 0;

out3 << cumulTime << "\t";

time_S1 = lambda_one*log(1/(1-randNumber()));

// time_S1 = time_S1_det + time_S1_ran;

mass = mass*exp(increment * time_S1);

out5 << mass << "\t";

cumulTime += time_S1;

state_Times.push_back(cumulTime); // time spent in state 1...

out3 << cumulTime << "\t";

CycA = calcCycA(CycA, 1, time_S1); // calculating Cyclin concentrations at the end of the state!!!

CycB = calcCycB(CycB, 1, time_S1); // calculating Cyclin concentrations at the end of the state!!!

CycE = calcCycE(CycE, 1, time_S1); // calculating Cyclin concentrations at the end of the state!!!

// CycD = calcCycD(CycD, time_S1);

out2 << CycA << "\t";

out6 << CycB << "\t";

out7 << CycE << "\t";

// out9 << CycD << "\t";

init_CycA_vec.push_back(CycA);

init_CycB_vec.push_back(CycB);

init_CycE_vec.push_back(CycE);

// init_CycD_vec.push_back(CycD);

if (CycE * mass >= threshold_two_three)

time_S2 = 0;

else

time_S2 = calcEarlyG1Time(kde_two, kse_two, increment, CycE, mass, init_t, threshold_two_three, out11); // early G1

cumulTime += time_S2;

mass = mass*exp(increment*time_S2); // mass at end of state 2, beginning of state 3

out5 << mass << "\t";

state_Times.push_back(cumulTime);

out3 << cumulTime << "\t";

CycA = calcCycA(CycA, 2, time_S2); // calculating Cyclin concentrations at the end of the state!!!

CycB = calcCycB(CycB, 2, time_S2); // calculating Cyclin concentrations at the end of the state!!!

CycE = calcCycE(CycE, 2, time_S2); // calculating Cyclin concentrations at the end of the state!!!

// CycD = calcCycD(CycD, time_S2);

// out11 << "Mass times the concentration of Cyclin E is: " << mass*CycE << endl;

out2 << CycA << "\t";

out6 << CycB << "\t";

out7 << CycE << "\t";

// out9 << CycD << "\t";

init_CycA_vec.push_back(CycA);

init_CycB_vec.push_back(CycB);

init_CycE_vec.push_back(CycE);

// init_CycD_vec.push_back(CycD);

if (CycA >= threshold_three_four_CycA)

{

time_S3_det = 0;

}

else

{

//time_S3_det = calcDetTime(CycA, kda_three, ksa_three); // was valid when trying to reach half-of-steady-state

time_S3_det = (double(1)/kda_three)*log((CycA - (ksa_three/kda_three))/((threshold_three_four_CycA - (ksa_three/kda_three))));

}

time_S3_ran = lambda_three*log(1/(1-randNumber()));

time_S3 = time_S3_det + time_S3_ran;

mass = mass*exp(increment*time_S3); // mass at end of state 3, beginning of state 4

out5 << mass << "\t";

cumulTime += time_S3;

state_Times.push_back(cumulTime);

out3 << cumulTime << "\t";

CycA = calcCycA(CycA, 3, time_S3); // calculating Cyclin concentrations at the end of the state!!!

CycB = calcCycB(CycB, 3, time_S3); // calculating Cyclin concentrations at the end of the state!!!

CycE = calcCycE(CycE, 3, time_S3); // calculating Cyclin concentrations at the end of the state!!!

// CycD = calcCycD(CycD, time_S3);

out2 << CycA << "\t";

out6 << CycB << "\t";

out7 << CycE << "\t";

// out9 << CycD << "\t";

init_CycA_vec.push_back(CycA);

init_CycB_vec.push_back(CycB);

init_CycE_vec.push_back(CycE);

// init_CycD_vec.push_back(CycD);

// Starting State 4 calculations

synthStartTime = cumulTime;

time_S4_det = 7;

time_S4_ran = lambda_four*log(1/(1-randNumber()));

time_S4 = time_S4_det + time_S4_ran;

synthDuration = time_S4;

cumulTime += time_S4;

mass = mass*exp(increment*time_S4); // mass at end of state 4, beginning of state 5

out5 << mass << "\t";

state_Times.push_back(cumulTime);

out3 << cumulTime << "\t";

CycA = calcCycA(CycA, 4, time_S4); // calculating Cyclin concentrations at the end of the state!!!

CycB = calcCycB(CycB, 4, time_S4); // calculating Cyclin concentrations at the end of the state!!!

CycE = calcCycE(CycE, 4, time_S4); // calculating Cyclin concentrations at the end of the state!!!

// CycD = calcCycD(CycD, time_S4);

out2 << CycA << "\t";

out6 << CycB << "\t";

out7 << CycE << "\t";

// out9 << CycD << "\t";

init_CycA_vec.push_back(CycA);

init_CycB_vec.push_back(CycB);

init_CycE_vec.push_back(CycE);

// init_CycD_vec.push_back(CycD);

synthStopTime = cumulTime;

// State 5 calculations

if (CycB >= threshold_five_six_CycB)

{

time_S5_det = 0;

}

else

{

time_S5_det = calcDetTime(CycB, kdb_five, ksb_five);

}

time_S5_ran = lambda_five*log(1/(1-randNumber()));

time_S5 = time_S5_det + time_S5_ran;

cumulTime += time_S5;

mass = mass*exp(increment*time_S5); // mass at end of state 5, beginning of state 6

out5 << mass << "\t";

state_Times.push_back(cumulTime);

out3 << cumulTime << "\t";

CycA = calcCycA(CycA, 5, time_S5); // calculating Cyclin concentrations at the end of the state!!!

CycB = calcCycB(CycB, 5, time_S5); // calculating Cyclin concentrations at the end of the state!!!

CycE = calcCycE(CycE, 5, time_S5); // calculating Cyclin concentrations at the end of the state!!!

// CycD = calcCycD(CycD, time_S5);

out2 << CycA << "\t";

out6 << CycB << "\t";

out7 << CycE << "\t";

// out9 << CycD << "\t";

init_CycA_vec.push_back(CycA);

init_CycB_vec.push_back(CycB);

init_CycE_vec.push_back(CycE);

// init_CycD_vec.push_back(CycD);

time_S6 = lambda_six*log(1/(1-randNumber())); // Prophase

cumulTime += time_S6;

mass = mass*exp(increment*time_S6); // mass at end of state 6, beginning of state 7

out5 << mass << "\t";

state_Times.push_back(cumulTime);

out3 << cumulTime << "\t";

CycA = calcCycA(CycA, 6, time_S6); // calculating Cyclin concentrations at the end of the state!!!

CycB = calcCycB(CycB, 6, time_S6); // calculating Cyclin concentrations at the end of the state!!!

CycE = calcCycE(CycE, 6, time_S6); // calculating Cyclin concentrations at the end of the state!!!

// CycD = calcCycD(CycD, time_S6);

out2 << CycA << "\t";

out6 << CycB << "\t";

out7 << CycE << "\t";

// out9 << CycD << "\t";

init_CycA_vec.push_back(CycA);

init_CycB_vec.push_back(CycB);

init_CycE_vec.push_back(CycE);

// init_CycD_vec.push_back(CycD);

time_S7 = lambda_seven*log(1/(1-randNumber())); // Prometaphase/Metaphase

cumulTime += time_S7;

mass = mass*exp(increment*time_S7); // mass at end of state 7, beginning of state 8

out5 << mass << "\t";

state_Times.push_back(cumulTime);

out3 << cumulTime << "\t";

CycA = calcCycA(CycA, 7, time_S7); // calculating Cyclin concentrations at the end of the state!!!

CycB = calcCycB(CycB, 7, time_S7); // calculating Cyclin concentrations at the end of the state!!!

CycE = calcCycE(CycE, 7, time_S7); // calculating Cyclin concentrations at the end of the state!!!

// CycD = calcCycD(CycD, time_S7);

out2 << CycA << "\t";

out6 << CycB << "\t";

out7 << CycE << "\t";

// out9 << CycD << "\t";

init_CycA_vec.push_back(CycA);

init_CycB_vec.push_back(CycB);

init_CycE_vec.push_back(CycE);

// init_CycD_vec.push_back(CycD);

time_S8 = lambda_eight*log(1/(1-randNumber())); // Anaphase

cumulTime += time_S8;

mass = mass*exp(increment*time_S8); // mass at end of state 8, beginning of state 9

out5 << mass << "\t";

state_Times.push_back(cumulTime);

out3 << cumulTime << "\t";

CycA = calcCycA(CycA, 8, time_S8); // calculating Cyclin concentrations at the end of the state!!!

CycB = calcCycB(CycB, 8, time_S8); // calculating Cyclin concentrations at the end of the state!!!

CycE = calcCycE(CycE, 8, time_S8); // calculating Cyclin concentrations at the end of the state!!!

// CycD = calcCycD(CycD, time_S8);

out2 << CycA << "\t";

out6 << CycB << "\t";

out7 << CycE << "\t";

// out9 << CycD << "\n";

init_CycA_vec.push_back(CycA);

init_CycB_vec.push_back(CycB);

init_CycE_vec.push_back(CycE);

// init_CycD_vec.push_back(CycD);

if (CycB <= threshold_nine_end)

{

time_S9_det = 0;

}

else

{

time_S9_det = (double(1)/kdb_nine)*log((CycB - (ksb_nine/kdb_nine))/((0.5*(ksb_eight/kdb_eight) - (ksb_nine/kdb_nine))));

}

time_S9_ran = lambda_nine*log(1/(1-randNumber()));

time_S9 = time_S9_det + time_S9_ran;

cumulTime += time_S9;

mass = mass*exp(increment*time_S9); // mass at end of state 9

out5 << mass << "\t";

state_Times.push_back(cumulTime);

out3 << cumulTime << "\n";

CycA = calcCycA(CycA, 9, time_S9); // calculating Cyclin concentrations at the end of the state!!!

CycB = calcCycB(CycB, 9, time_S9); // calculating Cyclin concentrations at the end of the state!!!

CycE = calcCycE(CycE, 9, time_S9); // calculating Cyclin concentrations at the end of the state!!!

// CycD = calcCycD(CycD, time_S9);

init_CycA_vec.push_back(CycA);

init_CycB_vec.push_back(CycB);

init_CycE_vec.push_back(CycE);

// init_CycD_vec.push_back(CycD);

out2 << CycA << "\n";

out6 << CycB << "\n";

out7 << CycE << "\n";

// out2 << CycA << "\t";

// out4 << mass << "\t";

mass = mass*0.5*box_muller(mean, deviation_mass);

out5 << mass << "\n";

cellCycleTime = cumulTime;

// out4 << cellCycleTime << endl;

if (j > 500)

{

phase = log(double(2/(2-randNumber())))/log(double(2));

timeChosen = phase*cellCycleTime;

out10 << timeChosen << "\t" << phase << "\t" << cellCycleTime << "\n";

for (int i = 0; i < state_Times.size(); i++)

{

flag = 0;

if (timeChosen <= state_Times[i])

{

flag = 1;

currState = i+1;

if (currState == 1) { initTime = 0; }

else if (currState > 1) { initTime = state_Times[i-1]; }

Amt_CycA = calcCycA(init_CycA_vec[i], currState, timeChosen-initTime)*(initial_mass*exp(increment*timeChosen));

Amt_CycB = calcCycB(init_CycB_vec[i], currState, timeChosen-initTime)*(initial_mass*exp(increment*timeChosen));

Amt_CycE = calcCycE(init_CycE_vec[i], currState, timeChosen-initTime)*(initial_mass*exp(increment*timeChosen));

// Amt_CycD = calcCycD(init_CycD_vec[i], timeChosen-initTime)*(initial_mass*exp(increment*timeChosen));

// out10 << j << endl;

Amt_CycA *= box_muller(mean, deviation_cyclin_a);

Amt_CycB *= box_muller(mean, deviation_cyclin_b);

Amt_CycE *= box_muller(mean, deviation_cyclin_e);

// Amt_CycD *= box_muller(mean, deviation_cyclin_d);

// measuring DNA content:

if (timeChosen <= synthStartTime)

{

DNA = 1;

}

else if (timeChosen >= synthStopTime)

{

DNA = 2;

}

else

{

DNA = 1 + (double(double(1)/synthDuration))*(timeChosen - synthStartTime);

}

DNA *= box_muller(mean, deviation_dna);

break;

}

}

out << currState << "\t" << DNA << "\t" << Amt_CycA << "\t" << Amt_CycE << "\t" << Amt_CycB << endl; // << "\t" << Amt_CycD << endl;

if (flag == 0) { cout << "WOOPS!!" << endl; }

// inserting the first 500 cells in the multimap one-by-one

clock_time_at_div = cellCycleTime - timeChosen;

Cell newCell;

newCell.cellNumber = cellNum;

newCell.clockTimeAtBgng = 0;

newCell.clockTimeAtDiv = clock_time_at_div;

newCell.cycleTime = cellCycleTime;

newCell.birthMass = birth_mass;

newCell.divMass = mass;

newCell.stateTimes = state_Times;

newCell.initCycAvec = init_CycA_vec;

newCell.initCycBvec = init_CycB_vec;

newCell.initCycEvec = init_CycE_vec;

// newCell.initCycDvec = init_CycD_vec;

cellPopulation.insert( pair<double,Cell>(clock_time_at_div, newCell) );

}

}

// outputting cells on Day 0

out13 << "Div. Clock Time\tCell #\tCycle Time" << endl;

for (cellIter = cellPopulation.begin(); cellIter != cellPopulation.end(); cellIter++)

{

out13 << cellIter->first << "\t" << cellIter->second.cellNumber << "\t" << cellIter->second.cycleTime << endl;

}

// processing cells after Day 0...

cellIter = cellPopulation.begin();

next_clock_time_at_div = cellIter->first;

int population_size;

double G0_trans_prob;

double G0_lambda;

double daught_birth_mass;

double mother_clock_time_at_div;

double daught1_time_at_div;

double daught2_time_at_div;

double CycA_from_mother, CycB_from_mother, CycE_from_mother;

bool end_flag;

int counter = 0;

int increment_counter;

int decrement_counter;

bool firstPass = true;

population_size = cellPopulation.size();

double thisBgngClockTime, thisDivClockTime;

while (next_clock_time_at_div < Hours) // checking that there are still cells that are going to divide before the simulation end time

{

Cell Daughter1;

Cell Daughter2;

CycA_from_mother = cellIter->second.initCycAvec.back();

CycB_from_mother = cellIter->second.initCycBvec.back();

CycE_from_mother = cellIter->second.initCycEvec.back();

counter++;

if (counter % 100 == 0) cout << counter << endl;

// GO LAMBDA CALCULATIONS...

daught_birth_mass = cellIter->second.divMass;

Daughter1.birthMass = daught_birth_mass;

Daughter2.birthMass = daught_birth_mass;

if (choice == 1)

G0_trans_prob = numerator/double(1+pow((double(population_size)/N_nought), double(Hill_Coeff)));

else

G0_trans_prob = numerator/double(1+exp((population_size - N_nought)/N_one)); // the one used

G0_lambda = double(1)/G0_trans_prob; // as described in the paper (G1a replaces G0)

out16 << G0_lambda << endl;

out17 << population_size << endl;

// the two daughters start off at the clock time of their parent's division

Daughter1.clockTimeAtBgng = next_clock_time_at_div;

Daughter2.clockTimeAtBgng = next_clock_time_at_div;

for (int d = 1; d <= 2; d++) // for simulating the two daughter cells

{

cellNum++;

// out4 << daught_birth_mass << "\t";

mass = daught_birth_mass;

init_CycA_vec.clear();

init_CycB_vec.clear();

init_CycE_vec.clear();

// init_CycD_vec.clear();

state_Times.clear();

init_CycA_vec.push_back(CycA_from_mother);

init_CycB_vec.push_back(CycB_from_mother);

init_CycE_vec.push_back(CycE_from_mother);

// init_CycD_vec.push_back(CycD);

// out5 << mass << "\t";

// out2 << CycA_from_mother << "\t";

// out6 << CycB_from_mother << "\t";

// out7 << CycE_from_mother << "\t";

// out9 << CycD << "\t";

cumulTime = 0;

// out3 << cumulTime << "\t";

time_S1 = G0_lambda*log(1/(1-randNumber()));

// time_S1 = time_S1_det + time_S1_ran;

mass = mass*exp(increment * time_S1);

// out5 << mass << "\t";

cumulTime += time_S1;

state_Times.push_back(cumulTime); // time spent in state 1...

// out3 << cumulTime << "\t";

CycA = calcCycA(CycA_from_mother, 1, time_S1); // calculating Cyclin concentrations at the end of the state!!!

CycB = calcCycB(CycB_from_mother, 1, time_S1); // calculating Cyclin concentrations at the end of the state!!!

CycE = calcCycE(CycE_from_mother, 1, time_S1); // calculating Cyclin concentrations at the end of the state!!!

// CycD = calcCycD(CycD, time_S1);

// out2 << CycA << "\t";

// out6 << CycB << "\t";

// out7 << CycE << "\t";

// out9 << CycD << "\t";

init_CycA_vec.push_back(CycA);

init_CycB_vec.push_back(CycB);

init_CycE_vec.push_back(CycE);

// init_CycD_vec.push_back(CycD);

if (CycE * mass >= threshold_two_three)

time_S2 = 0;

else

time_S2 = calcEarlyG1Time(kde_two, kse_two, increment, CycE, mass, init_t, threshold_two_three, out11); // early G1

cumulTime += time_S2;

mass = mass*exp(increment*time_S2); // mass at end of state 2, beginning of state 3

// out5 << mass << "\t";

state_Times.push_back(cumulTime);

// out3 << cumulTime << "\t";

CycA = calcCycA(CycA, 2, time_S2); // calculating Cyclin concentrations at the end of the state!!!

CycB = calcCycB(CycB, 2, time_S2); // calculating Cyclin concentrations at the end of the state!!!

CycE = calcCycE(CycE, 2, time_S2); // calculating Cyclin concentrations at the end of the state!!!

// CycD = calcCycD(CycD, time_S2);

// out11 << "Mass times the concentration of Cyclin E is: " << mass*CycE << endl;

// out2 << CycA << "\t";

// out6 << CycB << "\t";

// out7 << CycE << "\t";

// out9 << CycD << "\t";

init_CycA_vec.push_back(CycA);

init_CycB_vec.push_back(CycB);

init_CycE_vec.push_back(CycE);

// init_CycD_vec.push_back(CycD);

if (CycA >= threshold_three_four_CycA)

{

time_S3_det = 0;

}

else

{

//time_S3_det = calcDetTime(CycA, kda_three, ksa_three); // was valid when trying to reach half-of-steady-state

time_S3_det = (double(1)/kda_three)*log((CycA - (ksa_three/kda_three))/((threshold_three_four_CycA - (ksa_three/kda_three))));

}

time_S3_ran = lambda_three*log(1/(1-randNumber()));

time_S3 = time_S3_det + time_S3_ran;

mass = mass*exp(increment*time_S3); // mass at end of state 3, beginning of state 4

// out5 << mass << "\t";

cumulTime += time_S3;

state_Times.push_back(cumulTime);

// out3 << cumulTime << "\t";

CycA = calcCycA(CycA, 3, time_S3); // calculating Cyclin concentrations at the end of the state!!!

CycB = calcCycB(CycB, 3, time_S3); // calculating Cyclin concentrations at the end of the state!!!

CycE = calcCycE(CycE, 3, time_S3); // calculating Cyclin concentrations at the end of the state!!!

// CycD = calcCycD(CycD, time_S3);

// out2 << CycA << "\t";

// out6 << CycB << "\t";

// out7 << CycE << "\t";

// out9 << CycD << "\t";

init_CycA_vec.push_back(CycA);

init_CycB_vec.push_back(CycB);

init_CycE_vec.push_back(CycE);

// init_CycD_vec.push_back(CycD);

// Starting State 4 calculations

synthStartTime = cumulTime;

time_S4_det = 7;

time_S4_ran = lambda_four*log(1/(1-randNumber()));

time_S4 = time_S4_det + time_S4_ran;

synthDuration = time_S4;

cumulTime += time_S4;

mass = mass*exp(increment*time_S4); // mass at end of state 4, beginning of state 5

// out5 << mass << "\t";

state_Times.push_back(cumulTime);

// out3 << cumulTime << "\t";

CycA = calcCycA(CycA, 4, time_S4); // calculating Cyclin concentrations at the end of the state!!!

CycB = calcCycB(CycB, 4, time_S4); // calculating Cyclin concentrations at the end of the state!!!

CycE = calcCycE(CycE, 4, time_S4); // calculating Cyclin concentrations at the end of the state!!!

// CycD = calcCycD(CycD, time_S4);

// out2 << CycA << "\t";

// out6 << CycB << "\t";

// out7 << CycE << "\t";

// out9 << CycD << "\t";

init_CycA_vec.push_back(CycA);

init_CycB_vec.push_back(CycB);

init_CycE_vec.push_back(CycE);

// init_CycD_vec.push_back(CycD);

synthStopTime = cumulTime;

// State 5 calculations

if (CycB >= threshold_five_six_CycB)

{

time_S5_det = 0;

}

else

{

time_S5_det = calcDetTime(CycB, kdb_five, ksb_five);

}

time_S5_ran = lambda_five*log(1/(1-randNumber()));

time_S5 = time_S5_det + time_S5_ran;

cumulTime += time_S5;

mass = mass*exp(increment*time_S5); // mass at end of state 5, beginning of state 6

// out5 << mass << "\t";

state_Times.push_back(cumulTime);

// out3 << cumulTime << "\t";

CycA = calcCycA(CycA, 5, time_S5); // calculating Cyclin concentrations at the end of the state!!!

CycB = calcCycB(CycB, 5, time_S5); // calculating Cyclin concentrations at the end of the state!!!

CycE = calcCycE(CycE, 5, time_S5); // calculating Cyclin concentrations at the end of the state!!!

// CycD = calcCycD(CycD, time_S5);

// out2 << CycA << "\t";

// out6 << CycB << "\t";

// out7 << CycE << "\t";

// out9 << CycD << "\t";

init_CycA_vec.push_back(CycA);

init_CycB_vec.push_back(CycB);

init_CycE_vec.push_back(CycE);

// init_CycD_vec.push_back(CycD);

time_S6 = lambda_six*log(1/(1-randNumber())); // Prophase

cumulTime += time_S6;

mass = mass*exp(increment*time_S6); // mass at end of state 6, beginning of state 7

// out5 << mass << "\t";

state_Times.push_back(cumulTime);

// out3 << cumulTime << "\t";

CycA = calcCycA(CycA, 6, time_S6); // calculating Cyclin concentrations at the end of the state!!!

CycB = calcCycB(CycB, 6, time_S6); // calculating Cyclin concentrations at the end of the state!!!

CycE = calcCycE(CycE, 6, time_S6); // calculating Cyclin concentrations at the end of the state!!!

// CycD = calcCycD(CycD, time_S6);

// out2 << CycA << "\t";

// out6 << CycB << "\t";

// out7 << CycE << "\t";

// out9 << CycD << "\t";

init_CycA_vec.push_back(CycA);

init_CycB_vec.push_back(CycB);

init_CycE_vec.push_back(CycE);

// init_CycD_vec.push_back(CycD);

time_S7 = lambda_seven*log(1/(1-randNumber())); // Prometaphase/Metaphase

cumulTime += time_S7;

mass = mass*exp(increment*time_S7); // mass at end of state 7, beginning of state 8

// out5 << mass << "\t";

state_Times.push_back(cumulTime);

// out3 << cumulTime << "\t";

CycA = calcCycA(CycA, 7, time_S7); // calculating Cyclin concentrations at the end of the state!!!

CycB = calcCycB(CycB, 7, time_S7); // calculating Cyclin concentrations at the end of the state!!!

CycE = calcCycE(CycE, 7, time_S7); // calculating Cyclin concentrations at the end of the state!!!

// CycD = calcCycD(CycD, time_S7);

// out2 << CycA << "\t";

// out6 << CycB << "\t";

// out7 << CycE << "\t";

// out9 << CycD << "\t";

init_CycA_vec.push_back(CycA);

init_CycB_vec.push_back(CycB);

init_CycE_vec.push_back(CycE);

// init_CycD_vec.push_back(CycD);

time_S8 = lambda_eight*log(1/(1-randNumber())); // Anaphase

cumulTime += time_S8;

mass = mass*exp(increment*time_S8); // mass at end of state 8, beginning of state 9

// out5 << mass << "\t";

state_Times.push_back(cumulTime);

// out3 << cumulTime << "\t";

CycA = calcCycA(CycA, 8, time_S8); // calculating Cyclin concentrations at the end of the state!!!

CycB = calcCycB(CycB, 8, time_S8); // calculating Cyclin concentrations at the end of the state!!!

CycE = calcCycE(CycE, 8, time_S8); // calculating Cyclin concentrations at the end of the state!!!

// CycD = calcCycD(CycD, time_S8);

// out2 << CycA << "\t";

// out6 << CycB << "\t";

// out7 << CycE << "\t";

// out9 << CycD << "\n";

init_CycA_vec.push_back(CycA);

init_CycB_vec.push_back(CycB);

init_CycE_vec.push_back(CycE);

// init_CycD_vec.push_back(CycD);

if (CycB <= threshold_nine_end)

{

time_S9_det = 0;

}

else

{

// out3 << "viens" << CycB << "ici" << endl;

time_S9_det = (double(1)/kdb_nine)*log((CycB - (ksb_nine/kdb_nine))/((0.5*(ksb_eight/kdb_eight) - (ksb_nine/kdb_nine))));

}

time_S9_ran = lambda_nine*log(1/(1-randNumber()));

time_S9 = time_S9_det + time_S9_ran;

// time_S9 = lambda_nine*log(1/(1-randNumber()));

cumulTime += time_S9;

mass = mass*exp(increment*time_S9); // mass at end of state 9

// out5 << mass << "\t";

state_Times.push_back(cumulTime);

// out3 << cumulTime << "\n";

CycA = calcCycA(CycA, 9, time_S9); // calculating Cyclin concentrations at the end of the state!!!

CycB = calcCycB(CycB, 9, time_S9); // calculating Cyclin concentrations at the end of the state!!!

CycE = calcCycE(CycE, 9, time_S9); // calculating Cyclin concentrations at the end of the state!!!

// CycD = calcCycD(CycD, time_S9);

init_CycA_vec.push_back(CycA);

init_CycB_vec.push_back(CycB);

init_CycE_vec.push_back(CycE);

// out2 << CycA << "\n";

// out6 << CycB << "\n";

// out7 << CycE << "\n";

// out2 << CycA << "\t";

// out4 << mass << "\t";

mass = mass*0.5*box_muller(mean, deviation_mass);

// out5 << mass << "\n";

cellCycleTime = cumulTime;

// out4 << cellCycleTime << endl;

// record the parameters of the two spawned daughter cells

if (d == 1)

{

Daughter1.cellNumber = cellNum;

daught1_time_at_div = next_clock_time_at_div + cellCycleTime;

Daughter1.stateTimes = state_Times;

Daughter1.initCycAvec = init_CycA_vec;

Daughter1.initCycBvec = init_CycB_vec;

Daughter1.initCycEvec = init_CycE_vec;

Daughter1.divMass = mass;

}

else if (d == 2)

{

Daughter2.cellNumber = cellNum;

daught2_time_at_div = next_clock_time_at_div + cellCycleTime;

Daughter2.stateTimes = state_Times;

Daughter2.initCycAvec = init_CycA_vec;

Daughter2.initCycBvec = init_CycB_vec;

Daughter2.initCycEvec = init_CycE_vec;

Daughter2.divMass = mass;

}

}

Daughter1.clockTimeAtDiv = daught1_time_at_div;

Daughter2.clockTimeAtDiv = daught2_time_at_div;

cellPopulation.insert( pair<double, Cell>(daught1_time_at_div, Daughter1));

cellPopulation.insert( pair<double, Cell>(daught2_time_at_div, Daughter2));

cellIter++;

next_clock_time_at_div = cellIter->first;

population_size++;

}

int index = 0;

double bgngClockTime;

double divClockTime;

int Day10_popn = 0;

int Day0_popn = 0, Day1_popn = 0, Day2_popn = 0, Day3_popn = 0, Day4_popn = 0, Day5_popn = 0, Day6_popn = 0, Day7_popn = 0, Day8_popn = 0, Day9_popn = 0;

// outputting the number of cells present (population) on each successive day

// CELLS ARE SORTED ACCORDING TO THE TIME OF THEIR DIVISION, IN AN ASCENDING ORDER

for (cellIter = cellPopulation.begin(); cellIter != cellPopulation.end(); cellIter++)

{

index++;

bgngClockTime = cellIter->second.clockTimeAtBgng;

if (bgngClockTime <= Hours)

{

divClockTime = cellIter->first;

out14 << bgngClockTime << "\t" << index << endl;

out14 << divClockTime << "\t" << index << endl;

}

birth_mass = cellIter->second.birthMass;

state_Times = cellIter->second.stateTimes;

init_CycA_vec = cellIter->second.initCycAvec;

init_CycB_vec = cellIter->second.initCycBvec;

init_CycE_vec = cellIter->second.initCycEvec;

if (bgngClockTime <= 0)

{

Day0_popn++;

}

if (bgngClockTime <= 24 && divClockTime > 24)

{

outputFlowCytometry(24 - bgngClockTime, birth_mass, state_Times, init_CycA_vec, init_CycB_vec, init_CycE_vec, day1);

Day1_popn++;

}

if (bgngClockTime <= 48 && divClockTime > 48)

{

outputFlowCytometry(48 - bgngClockTime, birth_mass, state_Times, init_CycA_vec, init_CycB_vec, init_CycE_vec, day2);

Day2_popn++;

}

if (bgngClockTime <= 72 && divClockTime > 72)

{

outputFlowCytometry(72 - bgngClockTime, birth_mass, state_Times, init_CycA_vec, init_CycB_vec, init_CycE_vec, day3);

Day3_popn++;

}

if (bgngClockTime <= 96 && divClockTime > 96)

{

outputFlowCytometry(96 - bgngClockTime, birth_mass, state_Times, init_CycA_vec, init_CycB_vec, init_CycE_vec, day4);

Day4_popn++;

}

if (bgngClockTime <= 120 && divClockTime > 120)

{

outputFlowCytometry(120 - bgngClockTime, birth_mass, state_Times, init_CycA_vec, init_CycB_vec, init_CycE_vec, day5);

Day5_popn++;

}

if (bgngClockTime <= 144 && divClockTime > 144)

{

outputFlowCytometry(144 - bgngClockTime, birth_mass, state_Times, init_CycA_vec, init_CycB_vec, init_CycE_vec, day6);

Day6_popn++;

}

if (bgngClockTime <= 168 && divClockTime > 168)

{

outputFlowCytometry(168 - bgngClockTime, birth_mass, state_Times, init_CycA_vec, init_CycB_vec, init_CycE_vec, day7);

Day7_popn++;

}

if (bgngClockTime <= 192 && divClockTime > 192)

{

outputFlowCytometry(192 - bgngClockTime, birth_mass, state_Times, init_CycA_vec, init_CycB_vec, init_CycE_vec, day8);

Day8_popn++;

}

if (bgngClockTime <= 216 && divClockTime > 216)

{

outputFlowCytometry(216 - bgngClockTime, birth_mass, state_Times, init_CycA_vec, init_CycB_vec, init_CycE_vec, day9);

Day9_popn++;

}

if (bgngClockTime <= 240 && divClockTime > 240)

{

outputFlowCytometry(240 - bgngClockTime, birth_mass, state_Times, init_CycA_vec, init_CycB_vec, init_CycE_vec, day10);

Day10_popn++;

}

}

out15 << "Day\tPopulation" << endl;

out15 << "0\t" << Day0_popn << endl;

out15 << "1\t" << Day1_popn << endl;

out15 << "2\t" << Day2_popn << endl;

out15 << "3\t" << Day3_popn << endl;

out15 << "4\t" << Day4_popn << endl;

out15 << "5\t" << Day5_popn << endl;

out15 << "6\t" << Day6_popn << endl;

out15 << "7\t" << Day7_popn << endl;

out15 << "8\t" << Day8_popn << endl;

out15 << "9\t" << Day9_popn << endl;

out15 << "10\t" << Day10_popn << endl;

/// cout << "The population at the end of the simulation is: " << Day10_popn << endl;

}

void outputFlowCytometry(double timeChosen, double initial_mass, vector<double>& state_Times, vector<double>& init_CycA_vec, vector<double>& init_CycB_vec, vector<double> init_CycE_vec, ofstream &day )

{

double initTime;

int flag, currState;

double Amt_CycA, Amt_CycB, Amt_CycE;

double DNA;

float mean = 1;

float deviation_cyclin_a = 0.15;

float deviation_cyclin_b = 0.15;

float deviation_cyclin_e = 0.15;

// float deviation_cyclin_d = 0.2;

float deviation_dna = 0.03;

const double increment = 0.029;

// float deviation_mass = 0.033;

double synthStartTime = state_Times[3];

double synthStopTime = state_Times[4];

double synthDuration = synthStopTime - synthStartTime;

for (int i = 0; i < state_Times.size(); i++)

{

flag = 0;

if (timeChosen <= state_Times[i])

{

flag = 1;

currState = i+1;

if (currState == 1) { initTime = 0; }

else if (currState > 1) { initTime = state_Times[i-1]; }

Amt_CycA = calcCycA(init_CycA_vec[i], currState, timeChosen-initTime)*(initial_mass*exp(increment*timeChosen));

Amt_CycB = calcCycB(init_CycB_vec[i], currState, timeChosen-initTime)*(initial_mass*exp(increment*timeChosen));

Amt_CycE = calcCycE(init_CycE_vec[i], currState, timeChosen-initTime)*(initial_mass*exp(increment*timeChosen));

// Amt_CycD = calcCycD(init_CycD_vec[i], timeChosen-initTime)*(initial_mass*exp(increment*timeChosen));

// out10 << j << endl;

Amt_CycA *= box_muller(mean, deviation_cyclin_a);

Amt_CycB *= box_muller(mean, deviation_cyclin_b);

Amt_CycE *= box_muller(mean, deviation_cyclin_e);

// Amt_CycD *= box_muller(mean, deviation_cyclin_d);

// measuring DNA content:

if (timeChosen <= synthStartTime)

{

DNA = 1;

}

else if (timeChosen >= synthStopTime)

{

DNA = 2;

}

else

{

DNA = 1 + (double(double(1)/synthDuration))*(timeChosen - synthStartTime);

}

DNA *= box_muller(mean, deviation_dna);

break;

}

}

day << currState << "\t" << DNA << "\t" << Amt_CycA << "\t" << Amt_CycE << "\t" << Amt_CycB << endl; // << "\t" << Amt_CycD << endl;

if (flag == 0) { cout << "WOOPS!!" << endl; }

}

double calcCycA(double CycA, int state, double time)

{

int E2F, Cdc20_a, Cdh1, TF_b;

switch(state)

{

case 1:

// G0

E2F = 0;

TF_b = 0;

Cdc20_a = 0;

Cdh1 = 1;

break;

case 2:

// early G1

E2F = 1;

TF_b = 0;

Cdc20_a = 0;

Cdh1 = 1;

break;

case 3:

// late G1

E2F = 1;

TF_b = 0;

Cdc20_a = 0;

Cdh1 = 0;

break;

case 4:

// S

E2F = 1;

TF_b = 0;

Cdc20_a = 0;

Cdh1 = 0;

break;

case 5:

// G2

E2F = 1;

TF_b = 1;

Cdc20_a = 0;

Cdh1 = 0;

break;

case 6:

// Prophase

E2F = 0;

TF_b = 1;

Cdc20_a = 0;

Cdh1 = 0;

break;

case 7:

// Prometaphase/Metaphase

E2F = 0;

TF_b = 1;

Cdc20_a = 1;

Cdh1 = 0;

break;

case 8:

// Anaphase

E2F = 0;

TF_b = 1;

Cdc20_a = 1;

Cdh1 = 0;

break;

case 9:

// Telophase

E2F = 0;

TF_b = 0;

Cdc20_a = 1;

Cdh1 = 1;

break;

}

double k_sa = k_sa_p + k_sa_pp * E2F + k_sa_ppp * TF_b;

double k_da = k_da_p + k_da_pp * Cdc20_a + k_da_ppp * Cdh1;

CycA = (k_sa/k_da) + (CycA - (k_sa/k_da))*exp(-1*k_da*time);

return CycA;

}

/*

double calcCycD(double CycD, double time)

{

double k_sd = 0.2;

double k_dd = 0.2;

CycD = (k_sd/k_dd) + (CycD - (k_sd/k_dd))*exp(-1*k_dd*time);

return CycD;

}

*/

double calcCycB(double CycB, int state, double time)

{

int TF_b, Cdc20_b, Cdh1;

switch(state)

{

case 1:

// G0

TF_b = 0;

Cdc20_b = 0;

Cdh1 = 1;

break;

case 2:

// early G1

TF_b = 0;

Cdc20_b = 0;

Cdh1 = 1;

break;

case 3:

// late G1

TF_b = 0;

Cdc20_b = 0;

Cdh1 = 0;

break;

case 4:

// S

TF_b = 0;

Cdc20_b = 0;

Cdh1 = 0;

break;

case 5:

// G2

TF_b = 1;

Cdc20_b = 0;

Cdh1 = 0;

break;

case 6:

// Prophase

TF_b = 1;

Cdc20_b = 0;

Cdh1 = 0;

break;

case 7:

// Prometaphase/Metaphase

TF_b = 1;

Cdc20_b = 0;

Cdh1 = 0;

break;

case 8:

// Anaphase

TF_b = 1;

Cdc20_b = 1;

Cdh1 = 0;

break;

case 9:

// Telophase

TF_b = 0;

Cdc20_b = 1;

Cdh1 = 1;

break;

}

double k_sb = k_sb_p + k_sb_pp * TF_b;

double k_db = k_db_p + k_db_pp * Cdc20_b + k_db_ppp * Cdh1;

CycB = (k_sb/k_db) + (CycB - k_sb/k_db)*exp(-1*k_db*time);

return CycB;

}

double calcCycE(double CycE, int state, double time)

{

int E2F, SCF;

switch(state)

{

case 1:

// G0

E2F = 0;

SCF = 0;

break;

case 2:

// early G1

E2F = 1;

SCF = 0;

break;

case 3:

// late G1

E2F = 1;

SCF = 0;

break;

case 4:

// S

E2F = 1;

SCF = 1;

break;

case 5:

// G2

E2F = 1;

SCF = 1;

break;

case 6:

// Prophase

E2F = 0;

SCF = 1;

break;

case 7:

// Prometaphase/Metaphase

E2F = 0;

SCF = 1;

break;

case 8:

// Anaphase

E2F = 0;

SCF = 1;

break;

case 9:

// Telophase

E2F = 0;

SCF = 1;

break;

}

double k_se = k_se_p + k_se_pp * E2F;

double k_de = k_de_p + k_de_pp * SCF;

CycE = (k_se/k_de) + (CycE - k_se/k_de)*exp(-1*k_de*time);

return CycE;

}

double calcDetTime(double init_Cyc, double kd, double ks)

{

double detTime; // deterministic time spent in a particular state

double one = 1, two = 2;

detTime = (one/kd)*log(two*(one - (kd*init_Cyc/ks)));

return detTime;

}

double calcEarlyG1Time(double kde, double kse, double increment, double CycE, double mass, double t, double threshold, ofstream &out11)

{

// w/Exponential growth

double CycE_ss = kse/kde;

double neg_F_t, FP_t;

double delta_t = 0;

double abs_delta_t;

bool done = false;

int counter = 0;

// out11 << "=================================" << endl;

while (done == false)

{

counter++;

if (counter <= 100)

{

neg_F_t = (-1*CycE_ss) + (CycE_ss - CycE)*(exp(-1*kde*t)) + (threshold*exp(-1*increment*t)/mass);

FP_t = kde*(CycE_ss - CycE)*(exp(-1*kde*t)) + (threshold*increment*exp(-1*increment*t)/mass);

delta_t = neg_F_t/FP_t;

// out11 << delta_t << endl;

if (delta_t < 0) { abs_delta_t = -1*delta_t; } else {abs_delta_t = delta_t; }

if (abs_delta_t <= pow(double(10), double(-6)))

{

done = true;

}

else

{

t = t + delta_t;

done = false;

}

}

else break;

}

if (done == true)

return t;

else cout << "The 'mass times concentration' iterations failed to converge!!" << endl;

}

/* boxmuller.c Implements the Polar form of the Box-Muller

Transformation

(c) Copyright 1994, Everett F. Carter Jr.

Permission is granted by the author to use

this software for any application provided this

copyright notice is preserved.

ftp://ftp.taygeta.com/pub/c/boxmuller.c

// Note by Rajat Singhania: made some cosmetic changes of my own to produce the code below.

*/

float box_muller(float m, float s) /* mean m, standard deviation s */

{

float x1, x2, w, y1, y2;

do {

x1 = 2.0 * randNumber() - 1.0;

x2 = 2.0 * randNumber() - 1.0;

w = x1 * x1 + x2 * x2;

} while ( w >= 1.0 );

w = sqrt( (-2.0 * log( w ) ) / w );

y1 = x1 * w;

y2 = x2 * w;

float toReturn1 = m + y1 * s;

float toReturn2 = m + y2 * s;

if (toReturn1 > 0)

return toReturn1;

else if (toReturn2 > 0)

return toReturn2;

}

double randNumber()

{

unsigned int number;

double max = 1.0;

int err;

double value;

err = rand_s( &number );

if (err != 0)

{

cout << "The rand_s function failed!\n";

}

value = (((double) number) / ((double) UINT_MAX * max));

return value;

}
